# PacBio Full-Length and Illumina Transcriptomes of the Gill Reveal the Molecular Response of *Corbicula fluminea* under Aerial Exposure

**DOI:** 10.3390/ijms231911474

**Published:** 2022-09-29

**Authors:** Ting Zhang, Haibo Wen, Dongpo Xu, Guohua Lv, Yanfeng Zhou

**Affiliations:** Laboratory of Freshwater Fisheries and Germplasm Resources Utilization, Ministry of Agriculture and Rural Affairs, Freshwater Fisheries Research Center, Chinese Academy of Fishery Sciences, Wuxi 214081, China

**Keywords:** full-length transcriptome, *Corbicula fluminea*, autophagy, ferroptosis, metabolism

## Abstract

Air exposure is a common stress for *Corbicula fluminea*, an economically important freshwater shellfish consumed in China, during aquaculture and transportation. However, little is known about its molecular responses to air exposure. Therefore, this study used a combination of PacBio full-length and Illumina transcriptomes to investigate its molecular responses to air exposure. A total of 36,772 transcripts were obtained using PacBio sequencing. Structural analysis identified 32,069 coding sequences, 1906 transcription factors, 8873 simple sequence repeats, and 17,815 long non-coding RNAs. Subcellular localization analysis showed that most transcripts were located in the cytoplasm and nucleus. After 96-h of air exposure, 210 differentially expressed genes (DEGs) in the gill were obtained via Illumina sequencing. Among these DEGs, most of the genes related to glycolysis, tricarboxylic acid cycle, lipid metabolism, and amino acid metabolism were upregulated. Additionally, many DEGs associated with immunity, cytoskeleton reorganization, autophagy, and ferroptosis were identified. These findings indicated that metabolic strategy change, immune response, cytoskeleton reconstruction, autophagy, and ferroptosis might be the important mechanisms that *C. fluminea* use to cope with air exposure. This study will enrich the gene resources of *C. fluminea* and provide valuable data for studying the molecular mechanisms coping with air exposure in *C. fluminea* and other freshwater mollusks.

## 1. Introduction

Bivalve mollusks often face stress as a result of air exposure during ebb tide, harvesting, anhydrous transport, or other out-of-water operations. Air exposure is usually accompanied by desiccation, hypoxia, and starvation stress, and can lead to adverse effects, including oxidative stress injury [1], cell apoptosis [2], tissue water loss [3], energy homeostasis imbalance [4], retarded growth [5], poor muscle quality [6], and death [7]. Many bivalve mollusks can mobilize certain molecular mechanisms to cope with air exposure. Heat-shock proteins (HSPs) genes, antioxidant genes, anti-apoptotic genes, immune-related genes, and metabolism-related genes are involved in the adaption to air exposure or hypoxia in bivalve mollusks [8,9,10,11]. Although many studies have explored the molecular responses of bivalve mollusks to air exposure, most studies have focused on intertidal marine shellfish, such as *Mercenaria mercenaria* [12], *Ruditapes philippinarum* [13], *Crassostrea gigas* [14], and *Chlamys farreri* [15], and little research has focused on freshwater shellfish.

The Asian clam *Corbicula fluminea* is a kind of freshwater shellfish with high nutritional, medicinal, and ecological values, and it is widely distributed across freshwater ecosystems all over the world [16,17,18,19]. Over the years, the popularity of *C. fluminea* and its by-products has increased in Japan and Southeast Asian countries, and it has become an important export among aquatic products produced in China [20]. However, there are some challenges in the production of *C. fluminea*, especially regarding its transportation. To reduce costs, *C. fluminea* is usually transported without water. Such a transport method causes *C. fluminea* to suffer from air exposure. A previous study reported that air exposure can cause tissue water loss and even death in *C. fluminea* [21]. Therefore, elucidating the responses of *C. fluminea* to air exposure is important for aquaculture and transportation. Nevertheless, until now, little data on the responses of *C. fluminea* to air exposure have been available. 

With the rapid development of high-throughput sequencing, transcriptome sequencing (RNA-seq) technology has been widely used to investigate the molecular mechanisms behind bivalve mollusks’ responses to various environmental stresses [22,23,24]. Nevertheless, most prior studies have used second-generation RNA-seq, which limits the read length and accuracy of the transcript [25]. Third-generation RNA-seq is an emerging technology based on single-molecule real-time (SMRT) sequencing. This technique overcomes the defects of second-generation RNA-seq and can provide full-length transcripts while accurately identifying their complex structure and the subcellular localization of transcripts [26]. This technology is being increasingly applied to the study of aquatic animals. The full-length transcriptome has been used to investigate the molecular responses of *Coilia nasus* to salinity stress [27], of *Exopalaemon carinicauda* to thermal stress [28], of *Litopenaeus vannamei* to ammonia-N stress [29], and of *M. mercenaria* to air exposure [30]. However, to the best of our knowledge, the full-length transcriptome of *C. fluminea* has not been characterized or applied to explore the molecular mechanisms responding to environmental stresses.

The gill is the organ that is directly in contact with the external water environment, and it plays an important role in the respiration metabolism, osmotic regulation, detoxification, and immunity of aquatic animals. It is also the first tissue affected by environmental factors [31]. Therefore, in this study, we obtained the full-length transcriptome of the gill of the freshwater mollusk *C. fluminea* for the first time using PacBio sequencing and analyzed the functional annotation, structure, and subcellular localization of transcripts. These findings will enrich the literature on gene resources and make up for the gaps in the research on the full-length transcriptome of *C. fluminea*. On this basis, second-generation RNA-seq was used to explore the molecular responses of *C. fluminea* under air exposure. Our findings will provide valuable data to elucidate the molecular mechanisms that come into play in *C. fluminea* and other freshwater mollusks when responding to air exposure.

## 2. Results

### 2.1. PacBio Sequencing Data 

The PacBio sequencing data were submitted to the Sequence Read Archive (SRA) database (accession number: PRJNA838463). As shown in Table 1, 19,812,616 subreads were obtained. Then, a total of 525,513 circular consensus sequencing (CCS) reads with an average length of 2105.43 bp were obtained through the processing of subreads. A total of 475,249 full-length non-chimera (FLNC) sequences were identified after looking for two-end primers and the ploy(A) tail signals of the CCS sequences. After clustering, correction, and redundancy removal analysis of FLNC sequences, a total of 36,772 transcripts with an average length of 2063.52 bp were obtained. The length distribution of all transcripts is shown in Figure S1. The BUSCO analysis showed that the completeness of our transcriptome was about 62%, and the proportion of fragmented genes was as low as 1.2% (Figure S2).

### 2.2. Functional Annotation of Transcripts

**Figure 1:**
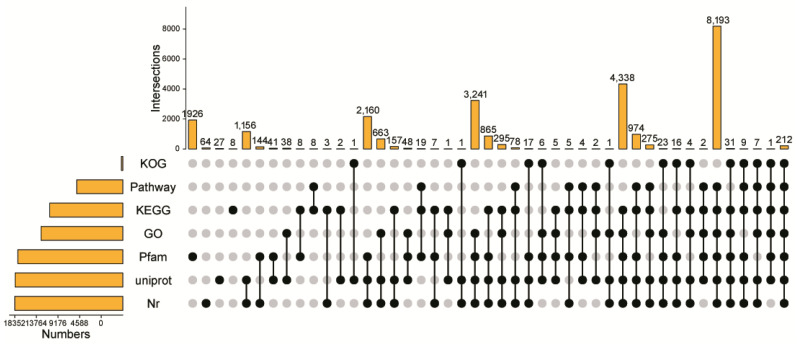
Upset Plot showing the numbers of transcripts annotated in seven databases.

Among the 36,772 transcripts, 25,086 were annotated across 7 databases. As shown in Figure 1, most of the transcripts were annotated in the Non-Redundant Protein Sequence Database (Nr) database (22,939), followed by annotation in the Unified Protein Database (Uniprot) database (22,894), the Protein families or domains (Pfam) database (22,295), the Gene Ontology (GO) database (17,388), the Kyoto Encyclopedia of Genes and Genomes (KEGG) database (15,529), the KEGG Pathway database (9789), and the Clusters of Eukaryotic Orthologous Groups (KOG) database (329). Of these, two-hundred and twelve transcripts were annotated across all seven databases. 

The Nr alignment results showed that the species with the highest sequence homology was *Pecten maximus*, followed by *C. gigas*, *Mizuhopecten yessoensis*, *Crassostrea virginica*, *Mytilus galloprovincialis*, *Mytilus coruscus*, etc., (Figure S3). GO classification analysis revealed that 22,304, 10,512, and 12,582 transcripts were included in the molecular function, biological process, and cellular component categories, respectively (Figure S4). KEGG analysis showed that most of the annotated transcripts were associated with the signal transduction pathway, followed by the pathways associated with the immune system, endocrine system, and global and overview metabolism maps (Figure S5). KOG analysis divided the function of the transcripts into 21 categories, with the majority of the transcripts being related to the function of the cytoskeleton and the signal transduction mechanism (Figure S6).

### 2.3. Structure and Subcellular Localization Analysis of Transcripts

**Figure 2:**
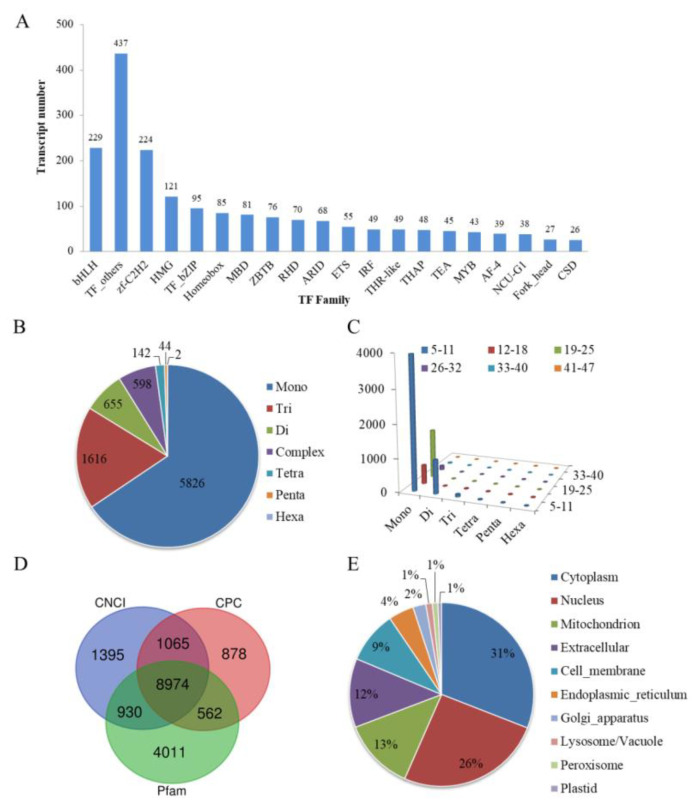
Structure analysis of transcripts. (**A**) Different families of transcript factors (TFs) were predicted by animalTFDB 3.0 software. (**B**,**C**) The classification and repetition numbers of simple repeat sequences (SSRs) were predicted by MISA 1.0 software. Mono: mono-nucleotide repetition; Di: di-nucleotide repetition: Tri: tri-nucleotide repetition; Tetra: tetra-nucleotide repetition; Penta: penta-nucleotide repetition; Hexa: hexa-nucleotide repetition; Complex: complex nucleotide repetition type. (**D**) The Venn diagram showed the numbers of long non-coding RNA (lncRNA) predicted using the CNCI, CPC, and Pfam databases. (**E**) Subcellular localization of transcripts was predicted by Deeploc software.

A total of 32,069 coding sequences (CDSs) were predicted using the TransDecoder software, and the N50 length of CDS was 1620 bp (Figure S7). The Animal Transcription Factor Database (animalTFDB) software predicted 1906 transcription factors (TFs) and the most TFs belonged to the bHLH and zf-C2H2 families (Figure 2A). The MISA software predicted 8873 simple repeat sequences (SSRs). The most predominant SSR type was the mono-nucleotide repetition type, followed by the tri-nucleotide repetition type (Figure 2B). The numbers of repetitions of most SSRs were in the range of 5–11, 19–25, and 12–18 (Figure 2C). A total of 14,477, 12,364, and 11,479 long noncoding RNAs (lncRNAs) were identified by the Pfam, Coding-Non-Coding Index (CNCI), and Coding Potential Calculator (CPC) databases, respectively, among which 8974 lncRNAs were identified from across all three databases (Figure 2D). The Deeploc software was used to predict the subcellular localization of the transcripts, most of which were located in the cytoplasm, nucleus, mitochondrion, extracellular, and cell membrane (Figure 2E).

### 2.4. Identification and Verification of DEGs

The Illumina sequencing data were submitted to the SRA database (accession number: PRJNA853077). As shown in Table 2, the clean bases of the six samples ranged from 5.26 G to 7.42 G. The ranges for Q20 and Q3 were 97.86–98.38% and 92.77–94.24%, respectively. 

**Figure 3:**
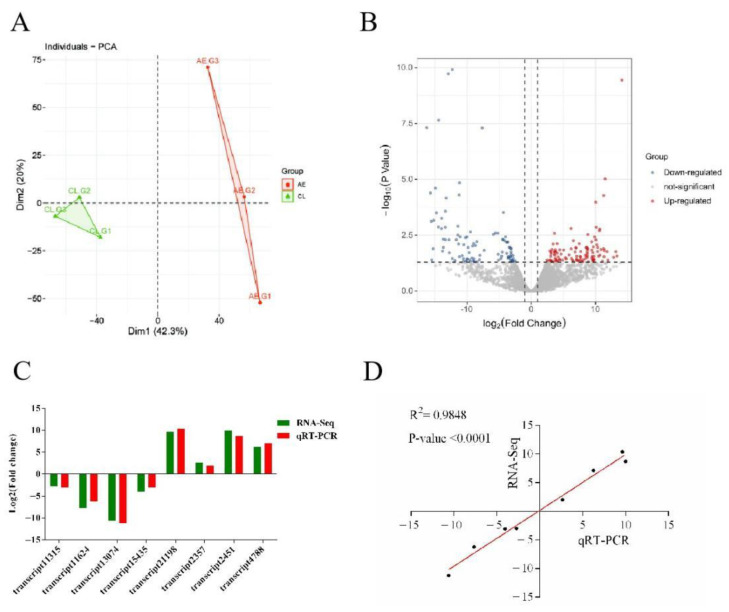
Identification and verification of DEGs. (**A**) The principal component analysis (PCA) of transcript expression in the control group (CL) and air exposure group (AE). (**B**) Volcano map of DEGs. (**C**) Comparison of the RNA-Seq and qRT-PCR results. (**D**) Correlation of gene expression results respectively obtained by RNA-Seq and qRT-PCR.

The principal component analysis (PCA) analysis of transcript expression showed that the samples in the control group (CL) and air exposure group (AE) were clustered separately (Figure 3A), suggesting significant differences in the transcriptomes between the two groups. A total of 210 differentially expressed genes (DEGs) were identified after air exposure, including 109 upregulated DEGs and 101 downregulated DEGs (Figure 3B). Four upregulated and four downregulated DEGs were randomly selected to verify the reliability of the transcriptome data using real-time quantitative PCR (qRT-PCR). As shown in Figure 3C, the variation trends in gene expression detected by qRT-PCR were consistent with those of RNA-seq. The linear regression analysis further showed a significant linear correlation and a positive correlation between RNA-seq and qRT-PCR (Figure 3D), which indicates that RNA-seq was reliable.

### 2.5. Analysis of DEGs

#### 2.5.1. GO and KEGG Enrichment Analysis of DEGs

**Figure 4:**
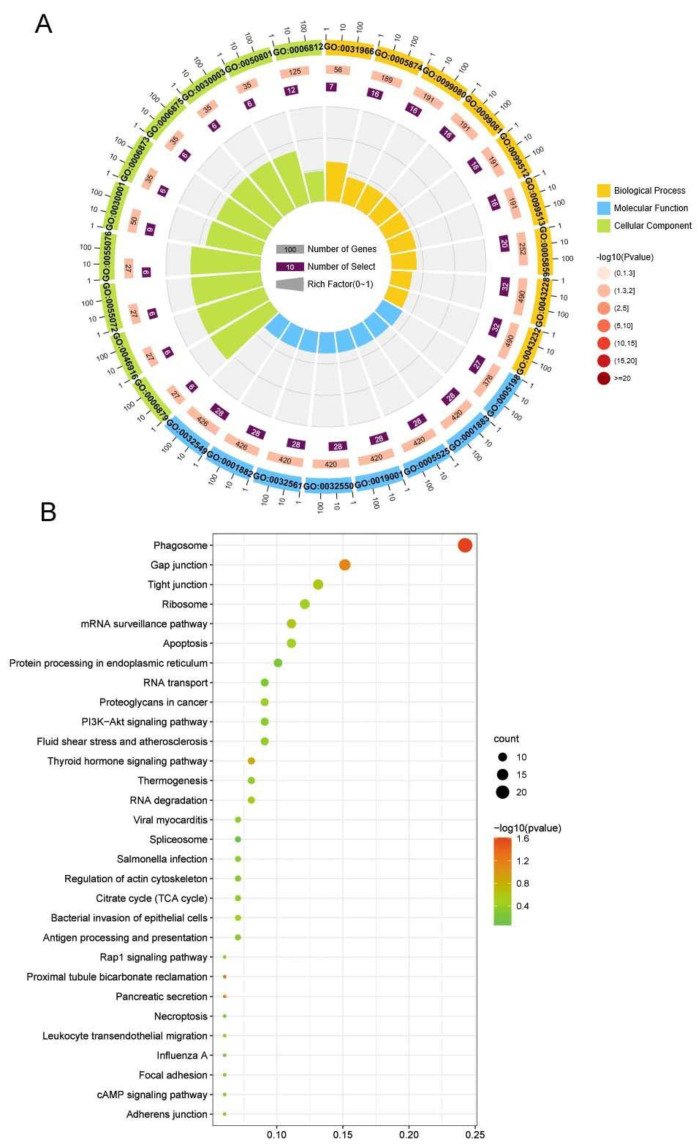
GO and KEGG enrichment analysis of DEGs. (**A**) The top 30 significantly enriched GO terms of DEGs. (**B**) The top 30 enriched KEGG pathways of DEGs.

To explore the potential functions of the DEGs, GO enrichment analysis was performed. As illustrated in Figure 4A and Table S1, the DEGs were enriched in three categories. For molecular function, the majority of the DEGs were enriched in the GO term of hydrolase activity (GO:0016787), followed by drug binding (GO:0008144). For the cellular component, the DEGs were mainly enriched in the GO terms of the non-membrane-bounded organelles (GO:0043228) and intracellular non-membrane-bounded organelles (GO:0043232). Regarding the biological processes, most of the DEGs were enriched in the GO term of metal ion transport (GO:0030001).

A KEGG enrichment analysis of the DEGs was conducted to further identify the effective pathways linked to air exposure. As shown in Figure 4B and Table S2, most of the DEGs were enriched in the phagosome pathway, followed by the gap junction and tight junction pathways. Additionally, some DEGs were enriched in the metabolism, immune, and cell death-associated pathways, such as the citrate cycle (TCA cycle), antigen processing and presentation, leukocyte transendothelial migration, and apoptosis.

#### 2.5.2. Identification of DEGs Associated with Metabolism

**Figure 5:**
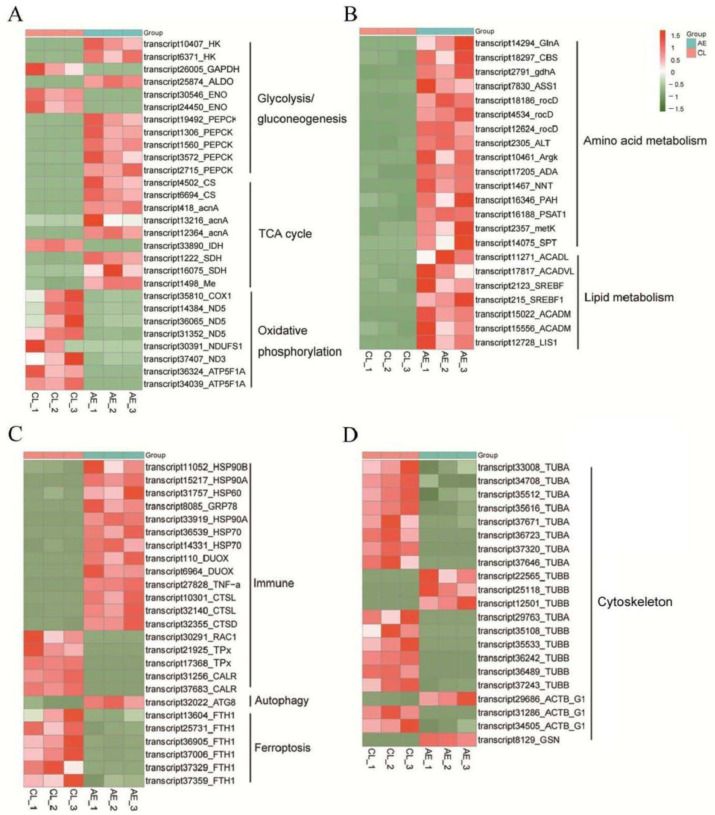
Heatmaps showed DEGs related to metabolism, immune, autophagy, ferroptosis, and cytoskeleton. (**A**) DEGs were related to glycolysis/gluconeogenesis, the TCA cycle, and oxidative phosphorylation. (**B**) DEGs were related to amino acid and lipid metabolism. (**C**) DEGs were related to immune, autophagy, and ferroptosis. (**D**) DEGs were related to the cytoskeleton. The color scale represents FPKM after standard normalization. *HK*: hexokinase; *Aldo*: fructose-bisphosphate aldolase; *GAPDH*: glyceraldehyde-3-phosphate dehydrogenase; *Eno*: enolase; *PEPCK*: phosphoenolpyruvate carboxykinase; *CS*: citrate synthase; *AcnA*: aconitate hydratase; *IDH*: Isocitrate dehydrogenase; *SDH*: succinate dehydrogenase; Me: malic enzyme. *COX1*: cytochrome c oxidase subunit I; *ND5/ ND3*: NADH dehydrogenase subunit 5/3; *NDUFS1*: ubiquinone oxidoreductase core subunit S1; ATP5F1A: ATP synthase F1 subunit alpha; *GlnA*: glutamine synthetase; *CBS*: cystathionine beta-synthase; gdhA: glutamate dehydrogenase; *ASS1*: argininosuccinate synthase 1; *rocD*: ornithine aminotransferase; *ALT*: alanine transaminase; *Argk*: Arginine kinase; *ADA*: adenosine deaminase; *NNT*: nicotinamide nucleotide transhydrogenase; *PAH*: phenylalanine hydroxylase; *PSAT1*: phosphoserine aminotransferase 1; *metK*: methionine adenosyltransferase; *SPT*: aminotransferase class I/II; *ACADL*: acyl-CoA dehydrogenase long chain; *ACADVL*: acyl-CoA dehydrogenase very long chain; *SREBF*: sterol regulatory element-binding protein; *ACADM*: acyl-CoA dehydrogenase medium-chain; *LIS1*: lissencephaly-1 homolog; *HSP90B*: heat-shock protein 90 alpha family class B member 1; *HSP90A*: heat-shock protein 90 alpha family class A member 1; *HSP60*: heat-shock protein 60; *GRP78*: glucose-regulated protein 78; *HSP70*: heat-shock protein 60; *DUOX*: dual oxidase; *CTSL*: cathepsin L; *CTSD*: cathepsin L; *TNF-α*: tumor necrosis factor; *TPx:* thioredoxin peroxidase; *Rac1*: rac family small GTPase 1; *CLAR*: calreticulin; *TUBA*: tubulin alpha chain; TUBB: tubulin beta chain; *FTH1*: ferritin heavy chain 1; *ATG8*: autophagy-associated protein; *ACTB_G1*: actin beta/gamma 1; *GSN*: gelsolin.

Based on the functional annotations of the transcripts, some DEGs related to metabolism were identified in this work. As shown in the heatmap in Figure 5A, most of the DEGs related to glycolysis/gluconeogenesis and the TCA cycle were upregulated after air exposure. These included *HK*, *Aldo*, *PEPCK*, *CS*, *AcnA*, and *SDH*. All eight of the DEGs involved in oxidative phosphorylation were downregulated after air exposure, including *COX1*, three *ND5* genes, *ND3*, *NDUFS1*, and two *ATP5F1A* genes. Furthermore, the genes associated with amino acid metabolism (*GlnA*, *CBS*, *gdhA*, *ASS1*, *rocD*, *ALT*, *ArgK*, *ADA*, *NNT*, *PAH*, *PAST1*, *metK*, and *SPT*) and lipid metabolism (*ACADL*, *ACADVL*, *SREBF*, *ACADM*, and *LIS1*) were significantly upregulated after air exposure (Figure 5B). These data indicate a changed metabolic strategy of *C. fluminea* in response to air exposure.

#### 2.5.3. Identification of DEGs Associated with Immunity

Several DEGs were linked to immunity, most of which were upregulated after air exposure, including many HSP genes (*HSP90A*, *HSP90B*, *HSP70*, *HSP60*, and *GRP78*), *CTSL*, *CTSD*, *DUOX2*, and *TNF-α*. However, the antioxidant-related gene (*TPx*) was downregulated after air exposure (Figure 5C). 

#### 2.5.4. Identification of DEGs Associated with the Cytoskeleton, Autophagy, and Ferroptosis

As shown in Figure 5C, *ATG8*, which is related to autophagy, was upregulated after air exposure, while the ferroptosis-related gene (*FTH1*) was downregulated after air exposure. In addition, the expression of many genes associated with the cytoskeleton, mainly actins and tubulins, changed after air exposure (Figure 5D).

## 3. Discussion

### 3.1. Full-Length Transcriptome of C. fluminea

In the past 10 years, several studies have reported on the second-generation transcriptome of *C. fluminea* [32,33,34], whereas none of the literature has reported on its full-length transcriptome. In this study, we analyzed the full-length transcriptome of *C. fluminea* using the PacBio RNA-seq method. We obtained 36,772 transcripts with an average length of 2105.43 bp, which was longer than 1263.53 bp, 859 bp, and 1690 bp that have been reported in prior studies [32,33,34]. We further analyzed the structure of the transcripts using various pieces of biological software. The animalTFDB 3.0 software predicted 1906 TFs and most of the TFs belonged to the bHLH and zf-C2H2 families, similar to a study on another shellfish, *Coelomactra antiquata* [35]. The MISA 1.0 software predicted 8873 SSRs, which was much more than the 2151 and 4279 SSRs reported by the second-generation RNA-seq [33,36]. The SSR types were mainly mono-nucleotide repeats and tri-nucleotide repeats, which is consistent with previous studies [33,36]. The Pfam, CNCI, and CPC databases were used to predict the coding potential of the transcripts, and 8974 lncRNAs were predicted by all three databases, suggesting that many of the transcripts may not have had protein-coding functions. In addition, the subcellular localization analysis revealed that more than half of the transcripts were located in the cytoplasm and nucleus, revealing that most of the transcripts may exert functions in these two organelles.

### 3.2. Molecular Responses to Air Exposure

**Figure 6:**
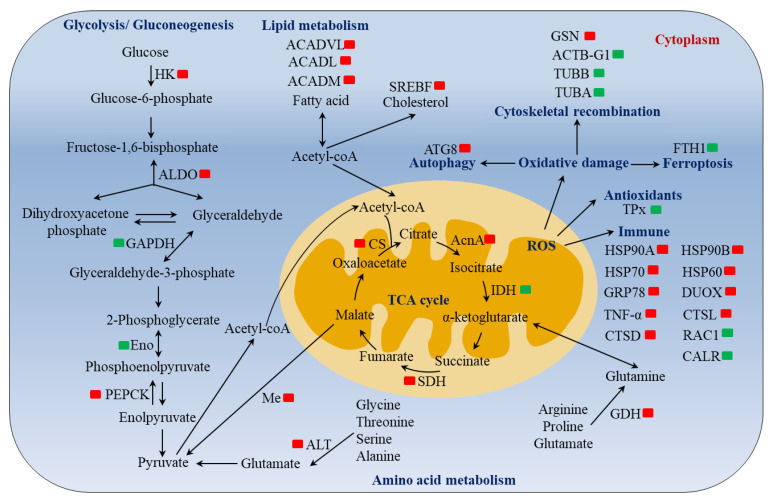
Schematic overview of air exposure-induced alterations of gene expression in metabolism, immune, cytoskeleton reconstruction, autophagy, and ferroptosis in the gill of *C. fluminea*. Red and green present upregulation and downregulation of genes, respectively.

RNA-seq technology has been widely used to investigate the molecular mechanisms of mollusks in response to air exposure and has been successfully used to study *M. mercenaria* [10,12], *R. philippinarum* [9], and *Solen grandis* [37]. *C. fluminea* is usually exposed to air during out-of-water operations, such as transportation or harvesting, but its molecular responses to air exposure remain largely unknown. In this study, we investigated the transcriptome changes in the gills of *C. fluminea* under 96 h of air exposure and identified 210 DEGs. The functional annotations showed that most of the DEGs in this study were related to metabolism, immune response, cytoskeletal reorganization, autophagy, and ferroptosis (Figure 6). 

#### 3.2.1. Molecular Responses in Metabolism

In general, the energy used by organisms is mainly provided through a series of metabolic pathways, such as glycolysis, the TCA cycle, and oxidative phosphorylation. A previous study showed that the expression of glycolysis/gluconeogenesis-related genes (*HK*, *PEPCK*, and *ALDO*) was upregulated during 96 h of air exposure in *C. gigas* and *Scylla paramamosain* [9,38]. Another study pointed out that the expression of TCA cycle-related genes (*CS* and *SDH*) were first decreased at the beginning (24 h) of hypoxia stress and then (96 h) increased in *Larimichthys crocea* [39]. Similarly, in this study, we also found some DEGs associated with glycolysis/gluconeogenesis, including *HK*, *PEPCK, ALDO*, *Eno*, and *GAPDH.* Among these, HK catalyzes the first step of glycolysis, which is one of the irreversible and rate-limiting steps of glycolysis. ALDO catalyzes the fourth step of glycolysis, which is a reversible reaction. Thus, the upregulations of HK and Aldo indicated that air exposure may have promoted the first and fourth steps of glycolysis. PEPCK catalyzes the rate-limiting reaction of gluconeogenesis, which can convert enolpyruvate, an intermediate product of glycolysis, to phosphoenolpyruvate. The upregulation of PEPCK in the present study may indicate that the conversion process of enolpyruvate to phosphoenolpyruvate was promoted. Eno and GAPDH catalyze the reversible reactions of glycolysis/gluconeogenesis. Therefore, we speculate that the downregulation of Eno and GAPDH may have occurred to avoid more glycolytic intermediates from producing glucose by gluconeogenesis. Furthermore, the genes associated with the TCA cycle (*CS*, *acnA*, *SDH*, and *Me*) were also upregulated after 96-h of air exposure. These results suggest that glycolysis products mainly entered the TCA cycle for aerobic metabolism to generate more energy. 

Under stress conditions caused by toxic substances or environmental factors, protein hydrolysis in aquatic animals releases free amino acids that are mobilized to provide additional energy requirements [40]. As described in previous research, after air exposure, alanine, glutamate, and other free amino acids in the plasma, liver, and muscle of *Paramisgurnus dabryanus* increased at different time points [41]. In addition, the expression of 26 genes related to amino acid metabolism increased after 96 h of hypoxic stress [39]. In this study, we also found that the expression of amino acid metabolism-associated genes, such as *ALT*, *gdhA*, and *GlnA*, was upregulated after 96 h of air exposure. Among them, the enzyme encoded by the *ALT* gene transfers amino groups from alanine to α-ketoglutarate, producing pyruvate and glutamate, which further enter the TCA cycle [42]. The enzyme encoded by the *gdhA* gene catalyzes the reversible reaction between α-ketoglutarate and glutamate, which is the first step for glutamate to enter the TCA cycle [43]. Thus, the upregulation of the *ALT* and *gdhA* genes indicated that air exposure promoted amino acid metabolism in the gills of *C. fluminea*, and certain amino acids entered the TCA cycle, providing additional energy for the organism. In addition, air exposure can lead to the accumulation of endogenous ammonia nitrogen in aquatic animals, which may result in ammonia toxicity [44]. It has been proven that glutamine synthesis plays an important role in ammonia tolerance in aquatic animals [41,45]. Therefore, in this study, the increase in *GlnA* expression may have helped the *C. fluminea* resist endogenous ammonia toxicity.

Aside from glucose and amino acids, lipids such as fatty acids and triglycerides are also important energy sources for organisms. A previous study showed that exposure to air induced the accumulation of four fatty acids in *Eriocheir sinensis*, which may be linked to the upregulation of the gene expression of fatty acid synthases [46]. In this study, we found that the expression of *ACADM*, *ACADL*, and *ADACVL* increased after air exposure. All three of these genes belong to the acyl-CoA dehydrogenase family, which catalyzes the first step of fatty acid β-oxidation [47]. Hence, we hypothesize that the β-oxidation of fatty acids was promoted under air exposure, providing more energy for *C. fluminea* to resist stress induced by air exposure. 

#### 3.2.2. Molecular Responses in Immune Systems

There is ample evidence that the immune systems of aquatic animals are sensitive to the stresses from various biological or abiotic factors, including air exposure [48]. The current study also identified many genes related to the immune system, such as the HSP genes, cathepsin (CTS) genes, proinflammatory cytokines, and antioxidant enzyme genes. HSPs are a class of molecular chaperones responsible for protecting proteins from misfolding and damage caused by cellular stress, and their increased expression is considered a key indicator of stress-induced protein damage [49]. In *E. sinensis*, the expression of *HSP70* and *HSP20* in the gills was upregulated after air exposure [50]. In *S. paramamosain*, the expression of *HSP90* and *HSP70* in the hepatopancreas was induced by air exposure [51]. In *Cherax quadricarinatus*, the expression of *HSP70*, *HSP90*, and *HSP21* increased during air exposure [52]. In our study, the expression of several HSPs, including *HSP90A*, *HSP90B*, *HSP60*, *HSP70*, and *GRP78*, was induced by air exposure, implying that HSPs play important roles in defending against air exposure of *C. fluminea*. TNF-α is a proinflammatory cytokine that exerts important functions in inflammation induction [53]. The upregulation of *TNF*-α in this study suggests that air exposure may have triggered inflammation in the gills of *C. fluminea*. CTSs are lysosomal enzymes that take part in many biological processes, including innate and adaptive immunity [54]. As previous studies have reported, the expression of CTSL was upregulated in *M. mercenaria* and *R. philippinarum* [12,37]. Likewise, this study also found an increase in the expression of *CTSL* and *CTSD* after air exposure, indicating the crucial role of CTS in the immunity of *C. fluminea*. TPx, as a member of the antioxidant enzymes, is important in maintaining the oxidation–reduction balance and protecting organisms from reactive oxygen species damage [55]. Thus, the downregulation of the *TPx* gene revealed that air exposure affected the antioxidant capacity of *C. fluminea*.

#### 3.2.3. Molecular Responses in Cytoskeletal Reorganization, Autophagy, and Ferroptosis

In this study, the gene expression of many cytoskeletal proteins, including actin and tubulin, altered after air exposure. Similarly, many cytoskeleton-associated genes were altered after 24-h of air exposure in *Pinctada fucata* [56], and 20-d of air exposure in *M. mercenaria* [12]. This evidence highlights the important role cytoskeletal reorganization plays in response to air exposure in aquatic animals. Programmed cell death (PCD) is an active and orderly way of inducing cell death initiated by gene regulation after stimulation from internal and external environmental factors [57]. The primary methods of PCD include apoptosis, autophagy, ferroptosis, and pyroptosis. Autophagy is generally regarded as a self-protection mechanism in response to adverse stimuli [58]. In our study, the expression of *ATG8*, a molecular marker of autophagy, was upregulated under air exposure. This finding suggests the activation of autophagy in the gills of *C. fluminea* under air exposure. Ferroptosis is a recently discovered form of non-apoptotic regulated cell death and can be caused by various stressors [59]. Previous studies have shown that heat stress or hypoxia can affect the expression of ferroptosis-related genes in aquatic animals [60,61]. Interestingly, we found that the expression of the *FTH1* gene decreased after air exposure. *FTH1* plays an important role in ferroptosis and its overexpression can inhibit the occurrence of ferroptosis [62]. Thus, the downregulation of the *FTH1* gene indicated that ferroptosis occurred in the gills of *C. fluminea* after air exposure. Taken together, the occurrences of autophagy and ferroptosis may be the self-protection mechanisms of *C. fluminea* in response to air exposure.

## 4. Materials and Methods

### 4.1. Animals and Air Exposure

The *C. fluminea* used in this study were provided by the Anhui Shuiyun Environmental Protection Technology Co., Ltd. (Wuhu, China). The morphological indices of *C. fluminea* were 3.80 ± 0.68 g in wet weight, 24.65 ± 1.32 mm in shell length, 20.25 ± 1.22 mm in shell height, and 12.92 ± 0.95 mm in shell width. Before the exposure experiment, all clams were kept in aerated water (dissolved oxygen: 7.79 ± 0.09 mg/L; pH: 8.0 ± 0.1; temperature: 20 ± 0.5 °C) for 2 weeks of temporary rearing and were fed twice daily with an algal suspension of cultivated *Chlorella vulgaris*. All animal procedures followed the guidelines of the Animal Experiments Ethics Committee of Freshwater Fisheries Research Center, Chinese Academy of Fishery Sciences.

After 2 weeks of temporary rearing, the clams were divided into CL and AE groups. Clams in the CL group were still kept in aerated water, and the feeding conditions were consistent with the temporary rearing period. Clams in the AE group were transferred to tanks without water. Three duplicate tanks were set up for each group. After 96 h, the gills of 15 clams from each group were collected, and immediately frozen in liquid nitrogen, and then stored at −80 °C for further transcriptomic analyses.

### 4.2. RNA Extraction

The total RNA was extracted from the gills using the Trizol reagent (Invitrogen, Carlsbad, CA, USA). The concentration and purity of the RNAs were determined by a NanoDrop spectrophotometer (Thermo Fisher Scientific, Waltham, MA, USA). RNA integrity was detected using the Agilent 2100 Bioanalyzer (Agilent Technologies, Sacramento, CA, USA), and only RNA samples with an RNA integrity number (RIN) > 7 were used for the cDNA library construction.

### 4.3. PacBio cDNA Library Construction and Third-Generation Sequencing

Equal amounts of total RNA from 18 gills (9 from the CL group and 9 from the AE group) were pooled to generate one sample. The mixed RNA sample was subjected to the synthesis of full-length cDNA using the SMARTer PCR cDNA Synthesis Kit (Clontech, San Francisco, CA, USA). The cDNA was used to construct an SMRT bell library with the DNA Template Prep Kit 2.0 (PacBio, San Francisco, CA, USA) and then sequenced on the Pacbio Sequal II platform at Magigene (Guangdong, China). 

### 4.4. PacBio Original Data Processing, Functional Annotation, and Structure Analysis

The PacBio raw sequencing data were processed with SMRT Link 5.1 software following the parameters: minLength = 200 and minReadScore = 0.65. First, reads containing adapters, reads containing poly-N, and reads with low quality (length < 50 bp, accuracy < 0.9) were removed from the original data to obtain subreads. Second, the subreads were self-corrected to generate CCS reads with parameters of min_length 200, max_drop_fraction 0.8, no_polish TRUE, min_zscore-9999, min_passes 1, min_predicted_accuracy 0.8, and max_length 18,000. Third, CCS reads containing 5′ primer, 3′ primer, and poly (A) tail signals were identified as FLNC and were identified as non-full-length sequences otherwise. Then, the isoseq3 cluster pipeline of the SMRT Link software was utilized to cluster the FLNC sequences to obtain consensus isoforms. Next, the Arrow software (parameters: hq_quiver_min_accuracy 0.99, bin_by_primer false, bin_size_kb 1, qv_trim_5p 100, qv_trim_3p 30) was used to polish the consensus reads. Finally, the redundant sequences in the polished consensus reads were removed using the CD-HIT software with a similarity threshold of 99%. The final obtained sequences were high-quality non-redundant full-length transcripts and were used for further analysis. The BUSCO Eukaryota v5.2.2_cv1 software was utilized to assess the completeness of transcriptome [63].

All transcripts were annotated in the Nr, KEGG, KOG, Pfam, GO, KEGG Pathway, and Uniprot databases by searching for the sequence or motif similarity. The Diamond BLASTX 2.0.6.144 software (parameter: e-value = 1 × 10^−5^) was used to perform the NR, KOG, Uniprot, GO, KEGG, and KEGG Pathway database analysis. The Hmmscan 3.3.2 software was used for family identification with the Pfam database. The CDSs were predicted using the TransDecoder software 5.5.0 (parameters: -m 50, -sin_best_only) according to the information on open reading frame length and log-likelihood score. TFs were identified and divided into different families by comparing transcripts against the animalTFDB 3.0 software. Transcripts with a length of >200 nucleotides were identified as lncRNAs and predicted using the CPC, CNCI, and Pfam analysis. The parameter used in CPC analysis was set to e-value = 1 × 10^−10^. The parameters in the CNCI analysis were the default ones. For Pfam analysis, the default parameters of –E 0.001 –domE 0.001 were used. Transcripts with coding potentials predicted by any or all of the above three tools were filtered out, and those transcripts without coding potential were candidate lncRNAs. SSRs were predicted using the MISA 1.0 software with default parameters. All SSRs were classified into seven types: mono-nucleotide repetition (Mono), di-nucleotide repetition (Di), tri-nucleotide repetition (Tri), tetra-nucleotide repetition (Tetra), penta-nucleotide repetition (Penta), hexa-nucleotide repetition (Hexa), and complex nucleotide repetition type (Complex) SSRs. The subcellular localization of the transcripts was analyzed using the Deeploc 1.0 software with default parameters. 

### 4.5. Illumina cDNA Library Construction and Second-Generation Sequencing 

Equal amounts of total RNA from three gills in the same group were pooled to generate one sample. Three mixed samples in the CL and AE groups, respectively, were obtained and used for the construction of the Illumina cDNA libraries. Then, 6 cDNA libraries (CL group: namely CL.G1, CL.G2, and CL.G3; AE group: namely AE.G1, AE.G2, and AE.G3) were sequenced on the Illumina HiSeqX10 platform at Magigene (Guangdong, China). The transcripts obtained by PacBio sequencing were used to establish a database as the reference sequences of the genes. Then, the clean reads obtained by Illumina sequencing were aligned to the established database using Bowtie2 software. The expression of the transcripts was calculated and normalized into the fragments per kilobase million (FPKM) mapped reads value using the RSEM 1.2.19 software [64]. The DEGs were identified by a threshold of false discovery rate (FDR) < 0.05 and |log2(fold change)| > 1. 

### 4.6. qRT-PCR Validation 

The Illumina RNA-seq results were verified by qRT-PCR. Four upregulated and four downregulated genes were randomly selected for qRT-PCR validation. The primers were designed using Primer 5.0 software and their sequence information is shown in Table S3. The elongation factor 1 alpha (EF1α) gene was utilized as a reference gene since our preliminary experiment showed that its expression did not respond to air exposure. The detailed experimental procedures were as previously described [65]. 

## 5. Conclusions

In summary, this study constructed a full-length transcriptome for the freshwater shellfish *C. fluminea* for the first time, and characterized the function, structure, and subcellular localization of transcripts, which enriched the gene resources and provided a basis for the molecular biology research of *C. fluminea*. Furthermore, this study explored the molecular responses of *C. fluminea* under air exposure using second-generation RNA-seq. Functional analysis of the DEGs suggested that metabolic strategy change, immune response, cytoskeleton reconstruction, and the activation of autophagy and ferroptosis might be important mechanisms used by *C. fluminea* to cope with air exposure (Figure 6). Our findings provide valuable data for understanding the molecular mechanisms used by *C. fluminea* and other freshwater mollusks in response to air exposure, as well as for the aquaculture and transportation of *C. fluminea*. Although some molecules that may be related to the response of *C. fluminea* to air exposure were found in this study, the specific functions of these molecules still need to be further explored. In addition, this study was performed at the transcriptome level. Thus, in the future, more omics methods, such as proteomics and metabolomic, may be integrated to better explore the molecular response of *C. fluminea* to air exposure.

## Figures and Tables

**Table 1:** The PacBio Sequencing Data.

Items	Seq Number	Seq Base (bp)	N50 (bp)	Max Length (bp)	Average Length (bp)	Min Length (bp)
Subreads	19,812,616	37,388,238,757	2211	258,357	1887.09	51
CCS reads	525,513	1,106,431,326	2312	13,047	2105.43	78
FLNC	475,249	944,431,109	2215	9867	1987.23	50
Cluster FLNC	38,161	78,712,507	2285	7555	2062.64	53
Transcripts	36,772	75,879,806	2290	7555	2063.52	53

**Table 2:** The Illumina sequencing data.

Sample	Clean Paired Reads	Clean Bases (G)	Q20 (%)	Q30 (%)	GC Content (%)	Clean Data Ratio (%)
CL.G1	23,509,678	6.80	98.26	93.79	38.33	88.78
CL.G2	25,615,479	7.42	98.21	93.67	37.02	88.5
CL.G3	23,849,453	6.91	97.86	92.77	35.18	87.23
AE.G1	23,828,332	6.85	98.38	94.23	41.00	88.96
AE.G2	23,980,738	6.94	98.38	94.24	40.38	89.40
AE.G3	18,229,077	5.26	98.27	93.95	40.57	88.82

## Data Availability

All of the data generated or analyzed during this study are included in this published article.

## Supplementary Materials

The supporting information can be downloaded at: https://www.mdpi.com/article/10.3390/ijms231911474/s1.
