# Supplementary material for: PacBio Full-Length and Illumina Transcriptomes of the Gill Reveal the Molecular Response of Corbicula fluminea under Aerial Exposure
Source: Int J Mol Sci. 2022 Sep 29;23(19):11474. doi: 10.3390/ijms231911474 (PMC9570311; doi:10.3390/ijms231911474)
Supplement: Supplementary file 1 [file ijms-23-11474-s001.zip › ijms-1831308-supplementary.pdf]

Supplementary Figures

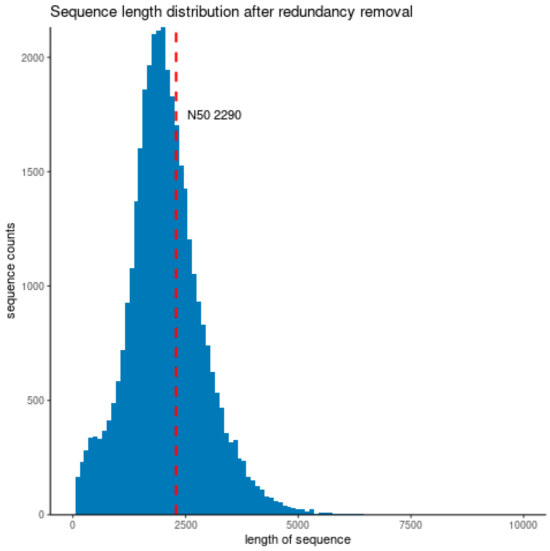

Figure S1. The length distribution of all transcripts.

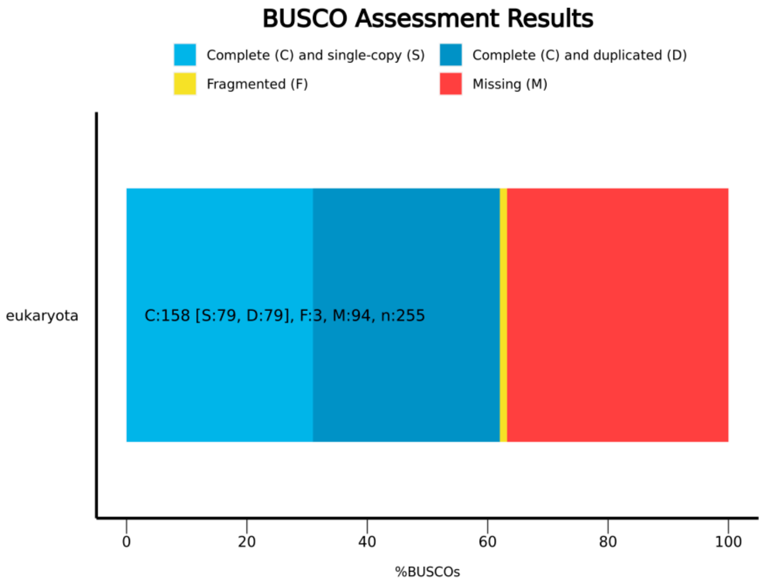

Figure S2. The completeness of the transcriptome was evaluated using BUSCO software.

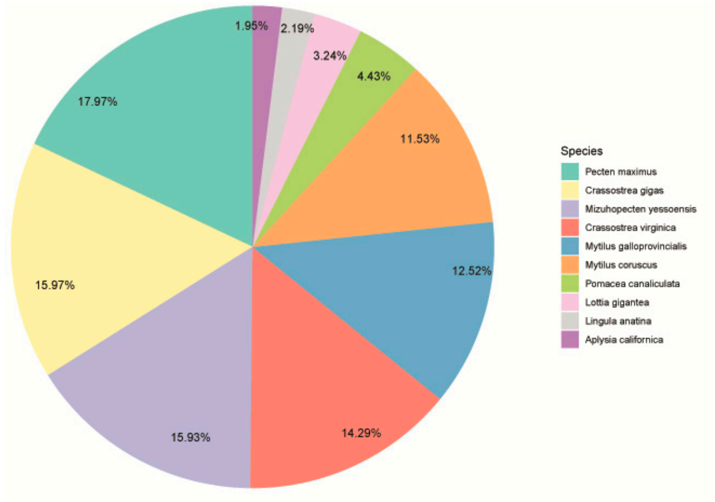

Figure S3. The dstribution of the top 10 species with matched transcripts in the Nr database.

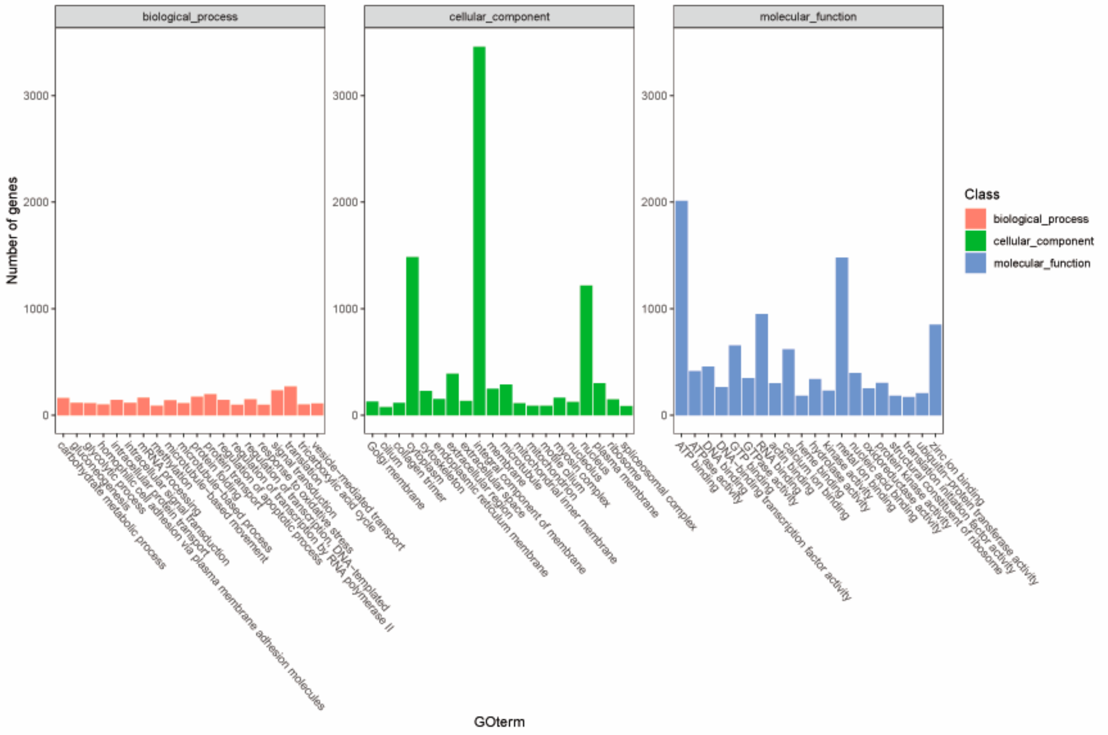

Figure S4. Distribution of GO terms for annotated transcripts.

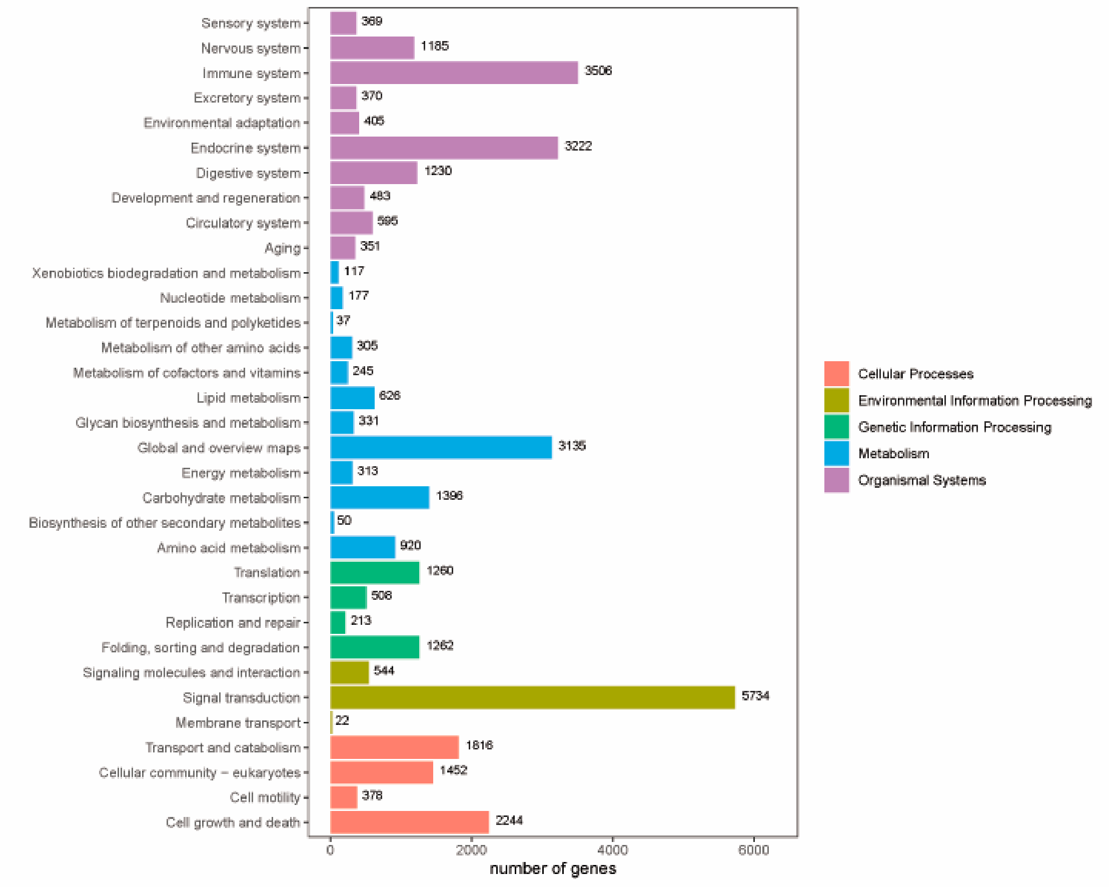

Figure S5. KEGG analysis of annotated transcripts.

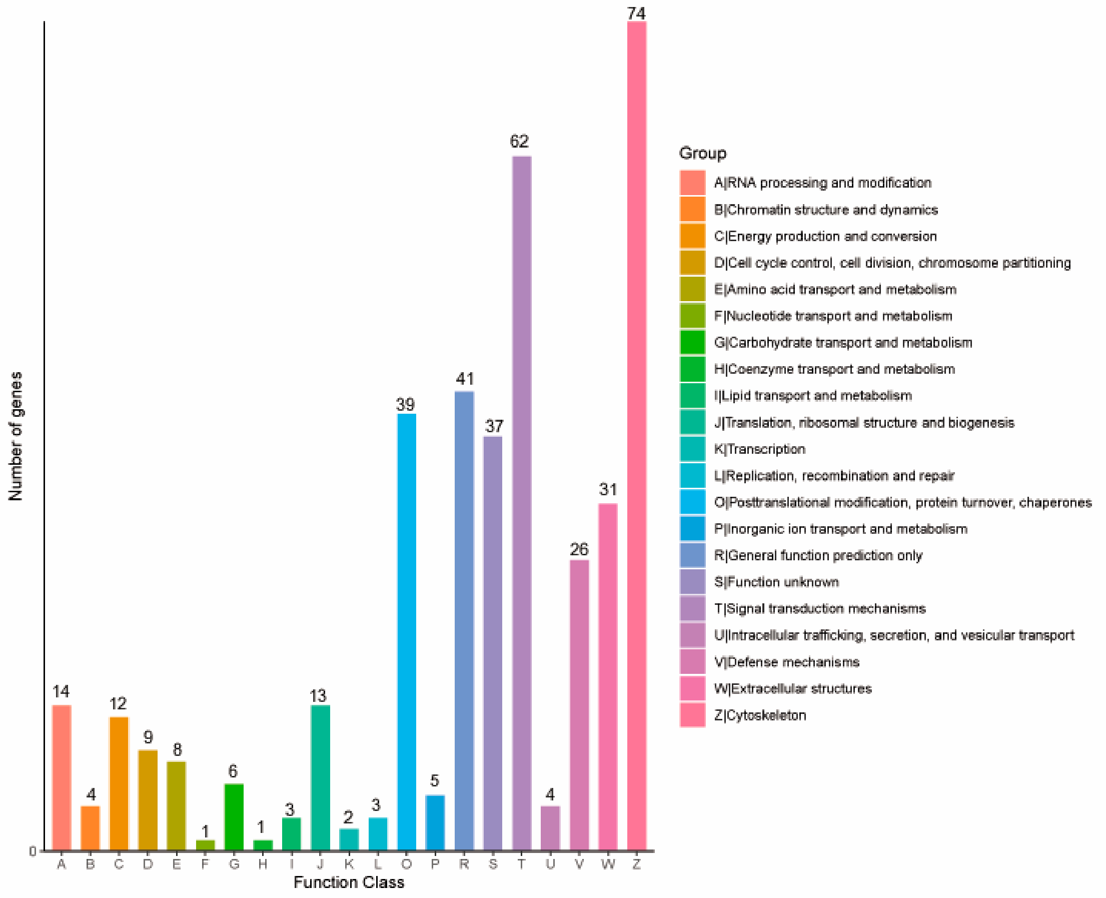

Figure S6. KOG analysis of annotated transcripts.

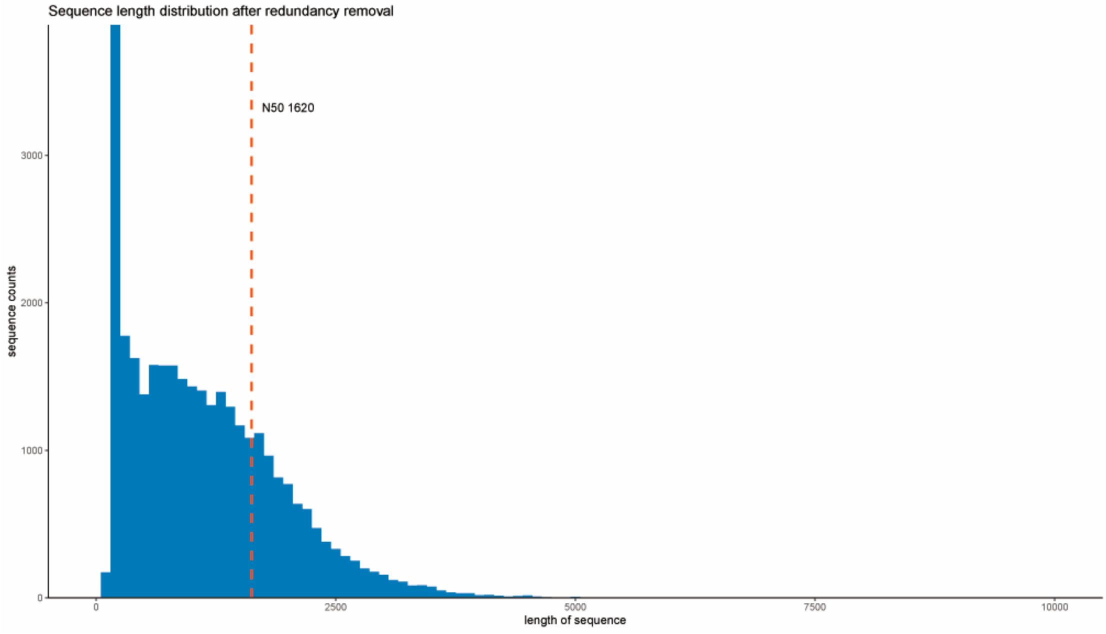

Figure S7. The length distribution of CDS.

Supplementary Tables

Table S1. GO enrichment analysis of DEGs.

GO ontology	GO ID	GO terms	Gene Ratio	BgRatio	q-value	Count
Biological Process	GO:0030001	metal ion transport	9/112	50/3002	0.023294	9
Biological Process	GO:0006879	cellular iron ion homeostasis	6/112	27/3002	0.024339	6
Biological Process	GO:0046916	cellular transition metal ion homeostasis	6/112	27/3002	0.024339	6
Biological Process	GO:0055072	iron ion homeostasis	6/112	27/3002	0.024339	6
Biological Process	GO:0055076	transition metal ion homeostasis	6/112	27/3002	0.024339	6
Biological Process	GO:0000041	transition metal ion transport	5/112	21/3002	0.037352	5
Biological Process	GO:0006826	iron ion transport	5/112	21/3002	0.037352	5
Biological Process	GO:0006873	cellular ion homeostasis	6/112	35/3002	0.037352	6
Biological Process	GO:0006875	cellular metal ion homeostasis	6/112	35/3002	0.037352	6
Biological Process	GO:0030003	cellular cation homeostasis	6/112	35/3002	0.037352	6
Biological Process	GO:0050801	ion homeostasis	6/112	35/3002	0.037352	6
Biological Process	GO:0055065	metal ion homeostasis	6/112	35/3002	0.037352	6
Biological Process	GO:0055080	cation homeostasis	6/112	35/3002	0.037352	6
Biological Process	GO:0098771	inorganic ion homeostasis	6/112	35/3002	0.037352	6
Biological Process	GO:0006812	cation transport	12/112	125/3002	0.044646	12
Biological Process	GO:0048878	chemical homeostasis	6/112	41/3002	0.068902	6
Biological Process	GO:0055082	cellular chemical homeostasis	6/112	41/3002	0.068902	6
Biological Process	GO:0006813	potassium ion transport	4/112	18/3002	0.068902	4
Biological Process	GO:0006811	ion transport	14/112	181/3002	0.113503	14
Biological Process	GO:0007010	cytoskeleton organization	11/112	138/3002	0.208549	11

Biological Process	GO:0042773	ATP synthesis coupled electron transport	3/112	15/3002	0.261245	3
Biological Process	GO:0006094	gluconeogenesis	7/112	76/3002	0.300392	7
Biological Process	GO:0019319	hexose biosynthetic process	7/112	76/3002	0.300392	7
Biological Process	GO:0046364	monosaccharide biosynthetic process	7/112	76/3002	0.300392	7
Biological Process	GO:0006378	mRNA polyadenylation	2/112	7/3002	0.318876	2
Biological Process	GO:0031124	mRNA 3'-end processing	2/112	7/3002	0.318876	2
Biological Process	GO:0007017	microtubule-based process	16/112	256/3002	0.318876	16
Biological Process	GO:0022904	respiratory electron transport chain	3/112	18/3002	0.321372	3
Biological Process	GO:0051234	establishment of localization	23/112	413/3002	0.321372	23
Biological Process	GO:0016051	carbohydrate biosynthetic process	7/112	82/3002	0.343031	7
Biological Process	GO:0031123	RNA 3'-end processing	2/112	8/3002	0.343031	2
Biological Process	GO:0051179	localization	23/112	421/3002	0.343031	23
Biological Process	GO:0006119	oxidative phosphorylation	1/112	1/3002	0.343031	1
Biological Process	GO:0009060	aerobic respiration	1/112	1/3002	0.343031	1
Biological Process	GO:0045333	cellular respiration	1/112	1/3002	0.343031	1
Biological Process	GO:0051321	meiotic cell cycle	1/112	1/3002	0.343031	1
Biological Process	GO:0043631	RNA polyadenylation	2/112	9/3002	0.36972	2
Biological Process	GO:0015672	monovalent inorganic cation transport	7/112	88/3002	0.36972	7
Biological Process	GO:0019725	cellular homeostasis	6/112	70/3002	0.36972	6
Biological Process	GO:0006810	transport	22/112	409/3002	0.36972	22
Biological Process	GO:0042592	homeostatic process	6/112	71/3002	0.380915	6
Biological Process	GO:0022900	electron transport chain	5/112	54/3002	0.385498	5

Biological Process	GO:0006836	neurotransmitter transport	2/112	10/3002	0.392955	2
Biological Process	GO:0009058	biosynthetic process	28/112	566/3002	0.462592	28
Biological Process	GO:1901576	organic substance biosynthetic process	27/112	545/3002	0.475322	27
Biological Process	GO:0006006	glucose metabolic process	7/112	97/3002	0.475322	7
Biological Process	GO:0019318	hexose metabolic process	7/112	97/3002	0.475322	7
Biological Process	GO:0006437	tyrosyl-tRNA aminoacylation	1/112	2/3002	0.475322	1
Biological Process	GO:0006888	endoplasmic reticulum to Golgi vesicle-mediated transport	1/112	2/3002	0.475322	1
Biological Process	GO:0006986	response to unfolded protein	1/112	2/3002	0.475322	1
Biological Process	GO:0035966	response to topologically incorrect protein	1/112	2/3002	0.475322	1
Biological Process	GO:0006412	translation	13/112	226/3002	0.476021	13
Biological Process	GO:0043043	peptide biosynthetic process	13/112	227/3002	0.480108	13
Biological Process	GO:0042221	response to chemical	2/112	13/3002	0.49596	2
Biological Process	GO:0043604	amide biosynthetic process	13/112	230/3002	0.49596	13
Biological Process	GO:0005996	monosaccharide metabolic process	7/112	102/3002	0.49596	7
Biological Process	GO:0006518	peptide metabolic process	13/112	232/3002	0.499586	13
Biological Process	GO:0006099	tricarboxylic acid cycle	5/112	64/3002	0.499586	5
Biological Process	GO:0034645	cellular macromolecule biosynthetic process	18/112	347/3002	0.499586	18
Biological Process	GO:0006996	organelle organization	12/112	211/3002	0.501975	12
Biological Process	GO:0043603	cellular amide metabolic process	13/112	235/3002	0.515451	13
Biological Process	GO:0000395	mRNA 5'-splice site recognition	1/112	3/3002	0.524908	1
Biological Process	GO:0006493	protein O-linked glycosylation	1/112	3/3002	0.524908	1
Biological Process	GO:0006900	vesicle budding from membrane	1/112	3/3002	0.524908	1

Biological Process	GO:0015980	energy derivation by oxidation of organic compounds	1/112	3/3002	0.524908	1
Biological Process	GO:0016050	vesicle organization	1/112	3/3002	0.524908	1
Biological Process	GO:0048194	Golgi vesicle budding	1/112	3/3002	0.524908	1
Biological Process	GO:0090114	COPII-coated vesicle budding	1/112	3/3002	0.524908	1
Biological Process	GO:0055114	oxidation-reduction process	15/112	290/3002	0.556217	15
Biological Process	GO:0009059	macromolecule biosynthetic process	18/112	361/3002	0.556217	18
Biological Process	GO:0055085	transmembrane transport	10/112	181/3002	0.556217	10
Biological Process	GO:0044283	small molecule biosynthetic process	7/112	116/3002	0.556217	7
Biological Process	GO:0000132	establishment of mitotic spindle orientation	1/112	4/3002	0.556217	1
Biological Process	GO:0009062	fatty acid catabolic process	1/112	4/3002	0.556217	1
Biological Process	GO:0040001	establishment of mitotic spindle localization	1/112	4/3002	0.556217	1
Biological Process	GO:0042026	protein refolding	1/112	4/3002	0.556217	1
Biological Process	GO:0042758	long-chain fatty acid catabolic process	1/112	4/3002	0.556217	1
Biological Process	GO:0051012	microtubule sliding	1/112	4/3002	0.556217	1
Biological Process	GO:0051017	actin filament bundle assembly	1/112	4/3002	0.556217	1
Biological Process	GO:0051293	establishment of spindle localization	1/112	4/3002	0.556217	1
Biological Process	GO:0051294	establishment of spindle orientation	1/112	4/3002	0.556217	1
Biological Process	GO:0051653	spindle localization	1/112	4/3002	0.556217	1
Biological Process	GO:0072329	monocarboxylic acid catabolic process	1/112	4/3002	0.556217	1
Biological Process	GO:1902850	microtubule cytoskeleton organization involved in mitosis	1/112	4/3002	0.556217	1
Biological Process	GO:1901566	organonitrogen compound biosynthetic process	15/112	301/3002	0.574223	15
Biological Process	GO:0065008	regulation of biological quality	7/112	121/3002	0.627439	7

Biological Process	GO:0006433	prolyl-tRNA aminoacylation	1/112	5/3002	0.637137	1
Biological Process	GO:0044242	cellular lipid catabolic process	1/112	5/3002	0.637137	1
Biological Process	GO:0051656	establishment of organelle localization	1/112	5/3002	0.637137	1
Biological Process	GO:0061572	actin filament bundle organization	1/112	5/3002	0.637137	1
Biological Process	GO:0006414	translational elongation	4/112	61/3002	0.689481	4
Biological Process	GO:0006101	citrate metabolic process	2/112	22/3002	0.689481	2
Biological Process	GO:0001676	long-chain fatty acid metabolic process	1/112	6/3002	0.689481	1
Biological Process	GO:0010035	response to inorganic substance	1/112	6/3002	0.689481	1
Biological Process	GO:0010038	response to metal ion	1/112	6/3002	0.689481	1
Biological Process	GO:0010039	response to iron ion	1/112	6/3002	0.689481	1
Biological Process	GO:0010040	response to iron(II) ion	1/112	6/3002	0.689481	1
Biological Process	GO:0051649	establishment of localization in cell	1/112	6/3002	0.689481	1
Biological Process	GO:0072350	tricarboxylic acid metabolic process	2/112	23/3002	0.705217	2
Biological Process	GO:0010033	response to organic substance	1/112	7/3002	0.766505	1
Biological Process	GO:0022414	reproductive process	1/112	7/3002	0.766505	1
Biological Process	GO:0006091	generation of precursor metabolites and energy	7/112	138/3002	0.817973	7
Biological Process	GO:0016042	lipid catabolic process	1/112	8/3002	0.817973	1
Biological Process	GO:0016054	organic acid catabolic process	1/112	8/3002	0.817973	1
Biological Process	GO:0044282	small molecule catabolic process	1/112	8/3002	0.817973	1
Biological Process	GO:0046395	carboxylic acid catabolic process	1/112	8/3002	0.817973	1
Biological Process	GO:0006457	protein folding	7/112	140/3002	0.817973	7
Biological Process	GO:0016999	antibiotic metabolic process	2/112	27/3002	0.817973	2

Biological Process	GO:0044271	cellular nitrogen compound biosynthetic process	15/112	338/3002	0.832867	15
Biological Process	GO:0007015	actin filament organization	2/112	28/3002	0.845373	2
Biological Process	GO:0006383	transcription by RNA polymerase III	1/112	9/3002	0.849834	1
Biological Process	GO:0061024	membrane organization	1/112	9/3002	0.849834	1
Biological Process	GO:1903047	mitotic cell cycle process	1/112	9/3002	0.849834	1
Biological Process	GO:0044281	small molecule metabolic process	17/112	395/3002	0.869143	17
Biological Process	GO:0007049	cell cycle	2/112	30/3002	0.889274	2
Biological Process	GO:0022402	cell cycle process	1/112	10/3002	0.895931	1
Biological Process	GO:0051640	organelle localization	1/112	10/3002	0.895931	1
Biological Process	GO:0005975	carbohydrate metabolic process	9/112	199/3002	0.9064	9
Biological Process	GO:0006376	mRNA splice site selection	1/112	11/3002	0.95202	1
Biological Process	GO:0097435	supramolecular fiber organization	2/112	33/3002	0.966965	2
Biological Process	GO:0044249	cellular biosynthetic process	19/112	461/3002	0.970617	19
Biological Process	GO:0006599	phosphagen metabolic process	1/112	12/3002	0.971541	1
Biological Process	GO:0006603	phosphocreatine metabolic process	1/112	12/3002	0.971541	1
Biological Process	GO:0042396	phosphagen biosynthetic process	1/112	12/3002	0.971541	1
Biological Process	GO:0046314	phosphocreatine biosynthetic process	1/112	12/3002	0.971541	1
Biological Process	GO:0000226	microtubule cytoskeleton organization	1/112	13/3002	0.99802	1
Biological Process	GO:0006366	transcription by RNA polymerase II	1/112	13/3002	0.99802	1
Biological Process	GO:0042398	cellular modified amino acid biosynthetic process	1/112	13/3002	0.99802	1
Biological Process	GO:0007264	small GTPase mediated signal transduction	2/112	36/3002	0.99802	2
Biological Process	GO:0006418	tRNA aminoacylation for protein translation	4/112	87/3002	0.99802	4

Biological Process	GO:0043038	amino acid activation	4/112	87/3002	0.99802	4
Biological Process	GO:0043039	tRNA aminoacylation	4/112	87/3002	0.99802	4
Biological Process	GO:0006575	cellular modified amino acid metabolic process	1/112	14/3002	0.99802	1
Biological Process	GO:0017144	drug metabolic process	3/112	64/3002	0.99802	3
Biological Process	GO:0006435	threonyl-tRNA aminoacylation	1/112	15/3002	0.99802	1
Biological Process	GO:0048193	Golgi vesicle transport	1/112	15/3002	0.99802	1
Biological Process	GO:0006399	tRNA metabolic process	4/112	91/3002	0.99802	4
Biological Process	GO:0016310	phosphorylation	12/112	301/3002	0.99802	12
Biological Process	GO:0000387	spliceosomal snRNP assembly	1/112	16/3002	0.99802	1
Biological Process	GO:0015698	inorganic anion transport	2/112	42/3002	0.99802	2
Biological Process	GO:0006605	protein targeting	1/112	17/3002	0.99802	1
Biological Process	GO:0006612	protein targeting to membrane	1/112	17/3002	0.99802	1
Biological Process	GO:0006613	cotranslational protein targeting to membrane	1/112	17/3002	0.99802	1
Biological Process	GO:0006614	SRP-dependent cotranslational protein targeting to membrane	1/112	17/3002	0.99802	1
Biological Process	GO:0045047	protein targeting to ER	1/112	17/3002	0.99802	1
Biological Process	GO:0072599	establishment of protein localization to endoplasmic reticulum	1/112	17/3002	0.99802	1
Biological Process	GO:0072657	protein localization to membrane	1/112	17/3002	0.99802	1
Biological Process	GO:0090150	establishment of protein localization to membrane	1/112	17/3002	0.99802	1
Biological Process	GO:1902600	proton transmembrane transport	3/112	70/3002	0.99802	3
Biological Process	GO:0006631	fatty acid metabolic process	1/112	18/3002	0.99802	1
Biological Process	GO:0051301	cell division	1/112	18/3002	0.99802	1
Biological Process	GO:0010638	positive regulation of organelle organization	1/112	19/3002	0.99802	1

Biological Process	GO:0030838	positive regulation of actin filament polymerization	1/112	19/3002	0.99802	1
Biological Process	GO:0031334	positive regulation of protein-containing complex assembly	1/112	19/3002	0.99802	1
Biological Process	GO:0032273	positive regulation of protein polymerization	1/112	19/3002	0.99802	1
Biological Process	GO:0034314	Arp2/3 complex-mediated actin nucleation	1/112	19/3002	0.99802	1
Biological Process	GO:0042176	regulation of protein catabolic process	1/112	19/3002	0.99802	1
Biological Process	GO:0044089	positive regulation of cellular component biogenesis	1/112	19/3002	0.99802	1
Biological Process	GO:0045010	actin nucleation	1/112	19/3002	0.99802	1
Biological Process	GO:0051495	positive regulation of cytoskeleton organization	1/112	19/3002	0.99802	1
Biological Process	GO:0070972	protein localization to endoplasmic reticulum	1/112	19/3002	0.99802	1
Biological Process	GO:1902905	positive regulation of supramolecular fiber organization	1/112	19/3002	0.99802	1
Biological Process	GO:0034660	ncRNA metabolic process	4/112	101/3002	0.99802	4
Biological Process	GO:0046034	ATP metabolic process	4/112	102/3002	0.99802	4
Biological Process	GO:0009894	regulation of catabolic process	1/112	20/3002	0.99802	1
Biological Process	GO:0006082	organic acid metabolic process	9/112	239/3002	0.99802	9
Biological Process	GO:0019752	carboxylic acid metabolic process	9/112	239/3002	0.99802	9
Biological Process	GO:0043436	oxoacid metabolic process	9/112	239/3002	0.99802	9
Biological Process	GO:0016043	cellular component organization	15/112	403/3002	0.99802	15
Biological Process	GO:0015985	energy coupled proton transport, down electrochemical gradient	1/112	22/3002	0.99802	1
Biological Process	GO:0015986	ATP synthesis coupled proton transport	1/112	22/3002	0.99802	1
Biological Process	GO:0051130	positive regulation of cellular component organization	1/112	22/3002	0.99802	1
Biological Process	GO:0006754	ATP biosynthetic process	1/112	23/3002	0.99802	1
Biological Process	GO:0006820	anion transport	2/112	52/3002	0.99802	2

Biological Process	GO:0071840	cellular component organization or biogenesis	15/112	415/3002	0.99802	15
Biological Process	GO:0009142	nucleoside triphosphate biosynthetic process	1/112	24/3002	0.99802	1
Biological Process	GO:0009144	purine nucleoside triphosphate metabolic process	1/112	24/3002	0.99802	1
Biological Process	GO:0009145	purine nucleoside triphosphate biosynthetic process	1/112	24/3002	0.99802	1
Biological Process	GO:0009199	ribonucleoside triphosphate metabolic process	1/112	24/3002	0.99802	1
Biological Process	GO:0009201	ribonucleoside triphosphate biosynthetic process	1/112	24/3002	0.99802	1
Biological Process	GO:0009205	purine ribonucleoside triphosphate metabolic process	1/112	24/3002	0.99802	1
Biological Process	GO:0009206	purine ribonucleoside triphosphate biosynthetic process	1/112	24/3002	0.99802	1
Biological Process	GO:0098660	inorganic ion transmembrane transport	4/112	112/3002	0.99802	4
Biological Process	GO:0098662	inorganic cation transmembrane transport	3/112	84/3002	0.99802	3
Biological Process	GO:0072594	establishment of protein localization to organelle	1/112	25/3002	0.99802	1
Biological Process	GO:0009141	nucleoside triphosphate metabolic process	1/112	26/3002	0.99802	1
Biological Process	GO:0006520	cellular amino acid metabolic process	4/112	115/3002	0.99802	4
Biological Process	GO:0006817	phosphate ion transport	1/112	27/3002	0.99802	1
Biological Process	GO:0033365	protein localization to organelle	1/112	27/3002	0.99802	1
Biological Process	GO:0034613	cellular protein localization	1/112	27/3002	0.99802	1
Biological Process	GO:0035435	phosphate ion transmembrane transport	1/112	27/3002	0.99802	1
Biological Process	GO:0044341	sodium-dependent phosphate transport	1/112	27/3002	0.99802	1
Biological Process	GO:0070727	cellular macromolecule localization	1/112	27/3002	0.99802	1
Biological Process	GO:0098655	cation transmembrane transport	3/112	89/3002	0.99802	3
Biological Process	GO:0044255	cellular lipid metabolic process	1/112	28/3002	0.99802	1
Biological Process	GO:0098661	inorganic anion transmembrane transport	1/112	28/3002	0.99802	1

Biological Process	GO:0050896	response to stimulus	2/112	62/3002	0.99802	2
Biological Process	GO:0006486	protein glycosylation	1/112	31/3002	0.99802	1
Biological Process	GO:0043413	macromolecule glycosylation	1/112	31/3002	0.99802	1
Biological Process	GO:0070085	glycosylation	1/112	31/3002	0.99802	1
Biological Process	GO:0008064	regulation of actin polymerization or depolymerization	1/112	33/3002	0.99802	1
Biological Process	GO:0030832	regulation of actin filament length	1/112	33/3002	0.99802	1
Biological Process	GO:0030833	regulation of actin filament polymerization	1/112	33/3002	0.99802	1
Biological Process	GO:0032271	regulation of protein polymerization	1/112	33/3002	0.99802	1
Biological Process	GO:0032535	regulation of cellular component size	1/112	33/3002	0.99802	1
Biological Process	GO:0032956	regulation of actin cytoskeleton organization	1/112	33/3002	0.99802	1
Biological Process	GO:0032970	regulation of actin filament-based process	1/112	33/3002	0.99802	1
Biological Process	GO:0043254	regulation of protein-containing complex assembly	1/112	33/3002	0.99802	1
Biological Process	GO:0044087	regulation of cellular component biogenesis	1/112	33/3002	0.99802	1
Biological Process	GO:0051493	regulation of cytoskeleton organization	1/112	33/3002	0.99802	1
Biological Process	GO:0090066	regulation of anatomical structure size	1/112	33/3002	0.99802	1
Biological Process	GO:0110053	regulation of actin filament organization	1/112	33/3002	0.99802	1
Biological Process	GO:1902903	regulation of supramolecular fiber organization	1/112	33/3002	0.99802	1
Biological Process	GO:0032787	monocarboxylic acid metabolic process	3/112	98/3002	0.99802	3
Biological Process	GO:0006793	phosphorus metabolic process	13/112	391/3002	0.99802	13
Biological Process	GO:0006796	phosphate-containing compound metabolic process	13/112	391/3002	0.99802	13
Biological Process	GO:0034220	ion transmembrane transport	4/112	129/3002	0.99802	4
Biological Process	GO:0090407	organophosphate biosynthetic process	2/112	70/3002	0.99802	2

Biological Process	GO:0009152	purine ribonucleotide biosynthetic process	1/112	36/3002	0.99802	1
Biological Process	GO:0009260	ribonucleotide biosynthetic process	1/112	36/3002	0.99802	1
Biological Process	GO:0046390	ribose phosphate biosynthetic process	1/112	36/3002	0.99802	1
Biological Process	GO:0006164	purine nucleotide biosynthetic process	1/112	37/3002	0.99802	1
Biological Process	GO:0072522	purine-containing compound biosynthetic process	1/112	37/3002	0.99802	1
Biological Process	GO:0033043	regulation of organelle organization	1/112	38/3002	0.99802	1
Biological Process	GO:0006397	mRNA processing	3/112	107/3002	0.99802	3
Biological Process	GO:0006629	lipid metabolic process	1/112	40/3002	0.99802	1
Biological Process	GO:1901137	carbohydrate derivative biosynthetic process	1/112	40/3002	0.99802	1
Biological Process	GO:0016071	mRNA metabolic process	3/112	110/3002	0.99802	3
Biological Process	GO:0006096	glycolytic process	2/112	77/3002	0.99802	2
Biological Process	GO:0006413	translational initiation	2/112	77/3002	0.99802	2
Biological Process	GO:0006757	ATP generation from ADP	2/112	77/3002	0.99802	2
Biological Process	GO:0009135	purine nucleoside diphosphate metabolic process	2/112	77/3002	0.99802	2
Biological Process	GO:0009179	purine ribonucleoside diphosphate metabolic process	2/112	77/3002	0.99802	2
Biological Process	GO:0009185	ribonucleoside diphosphate metabolic process	2/112	77/3002	0.99802	2
Biological Process	GO:0046031	ADP metabolic process	2/112	77/3002	0.99802	2
Biological Process	GO:0006351	transcription, DNA-templated	1/112	41/3002	0.99802	1
Biological Process	GO:0097659	nucleic acid-templated transcription	1/112	41/3002	0.99802	1
Biological Process	GO:0006090	pyruvate metabolic process	2/112	78/3002	0.99802	2
Biological Process	GO:0098656	anion transmembrane transport	1/112	42/3002	0.99802	1
Biological Process	GO:0009150	purine ribonucleotide metabolic process	3/112	113/3002	0.99802	3

Biological Process	GO:0009259	ribonucleotide metabolic process	3/112	113/3002	0.99802	3
Biological Process	GO:0019693	ribose phosphate metabolic process	3/112	113/3002	0.99802	3
Biological Process	GO:0006355	regulation of transcription, DNA-templated	5/112	178/3002	0.99802	5
Biological Process	GO:0051128	regulation of cellular component organization	1/112	43/3002	0.99802	1
Biological Process	GO:0006165	nucleoside diphosphate phosphorylation	2/112	81/3002	0.99802	2
Biological Process	GO:0009132	nucleoside diphosphate metabolic process	2/112	81/3002	0.99802	2
Biological Process	GO:0035556	intracellular signal transduction	2/112	81/3002	0.99802	2
Biological Process	GO:0046939	nucleotide phosphorylation	2/112	81/3002	0.99802	2
Biological Process	GO:0006163	purine nucleotide metabolic process	3/112	116/3002	0.99802	3
Biological Process	GO:0072521	purine-containing compound metabolic process	3/112	116/3002	0.99802	3
Biological Process	GO:0016052	carbohydrate catabolic process	2/112	82/3002	0.99802	2
Biological Process	GO:0034641	cellular nitrogen compound metabolic process	23/112	715/3002	0.99802	23
Biological Process	GO:0044267	cellular protein metabolic process	16/112	517/3002	0.99802	16
Biological Process	GO:1903506	regulation of nucleic acid-templated transcription	5/112	188/3002	0.99802	5
Biological Process	GO:2001141	regulation of RNA biosynthetic process	5/112	188/3002	0.99802	5
Biological Process	GO:2000112	regulation of cellular macromolecule biosynthetic process	5/112	190/3002	0.99802	5
Biological Process	GO:0019219	regulation of nucleobase-containing compound metabolic process	5/112	191/3002	0.99802	5
Biological Process	GO:0051252	regulation of RNA metabolic process	5/112	191/3002	0.99802	5
Biological Process	GO:0006950	response to stress	1/112	50/3002	0.99802	1
Biological Process	GO:0009165	nucleotide biosynthetic process	1/112	51/3002	0.99802	1
Biological Process	GO:0022618	ribonucleoprotein complex assembly	1/112	51/3002	0.99802	1
Biological Process	GO:0071826	ribonucleoprotein complex subunit organization	1/112	51/3002	0.99802	1

Biological Process	GO:1901293	nucleoside phosphate biosynthetic process	1/112	51/3002	0.99802	1
Biological Process	GO:0007165	signal transduction	3/112	126/3002	0.99802	3
Biological Process	GO:0051641	cellular localization	3/112	126/3002	0.99802	3
Biological Process	GO:0019637	organophosphate metabolic process	4/112	162/3002	0.99802	4
Biological Process	GO:1901135	carbohydrate derivative metabolic process	3/112	128/3002	0.99802	3
Biological Process	GO:0000375	RNA splicing, via transesterification reactions	1/112	53/3002	0.99802	1
Biological Process	GO:0000377	RNA splicing, via transesterification reactions with bulged adenosine as nucleophile	1/112	53/3002	0.99802	1
Biological Process	GO:0000398	mRNA splicing, via spliceosome	1/112	53/3002	0.99802	1
Biological Process	GO:0010468	regulation of gene expression	5/112	198/3002	0.99802	5
Biological Process	GO:0006886	intracellular protein transport	2/112	94/3002	0.99802	2
Biological Process	GO:0065007	biological regulation	16/112	539/3002	0.99802	16
Biological Process	GO:0009889	regulation of biosynthetic process	5/112	200/3002	0.99802	5
Biological Process	GO:0010556	regulation of macromolecule biosynthetic process	5/112	200/3002	0.99802	5
Biological Process	GO:0031326	regulation of cellular biosynthetic process	5/112	200/3002	0.99802	5
Biological Process	GO:0016070	RNA metabolic process	8/112	298/3002	0.99802	8
Biological Process	GO:0006753	nucleoside phosphate metabolic process	3/112	133/3002	0.99802	3
Biological Process	GO:0009117	nucleotide metabolic process	3/112	133/3002	0.99802	3
Biological Process	GO:0044260	cellular macromolecule metabolic process	21/112	693/3002	0.99802	21
Biological Process	GO:0055086	nucleobase-containing small molecule metabolic process	3/112	136/3002	0.99802	3
Biological Process	GO:0032774	RNA biosynthetic process	1/112	58/3002	0.99802	1
Biological Process	GO:0051246	regulation of protein metabolic process	1/112	60/3002	0.99802	1
Biological	GO:000639	RNA processing	3/112	143/300	0.99802	3

Process	6			2		
Biological Process	GO:0051171	regulation of nitrogen compound metabolic process	6/112	247/3002	0.99802	6
Biological Process	GO:0080090	regulation of primary metabolic process	6/112	247/3002	0.99802	6
Biological Process	GO:0048518	positive regulation of biological process	1/112	63/3002	0.99802	1
Biological Process	GO:0048522	positive regulation of cellular process	1/112	63/3002	0.99802	1
Biological Process	GO:0019538	protein metabolic process	19/112	649/3002	0.99802	19
Biological Process	GO:1901575	organic substance catabolic process	3/112	145/3002	0.99802	3
Biological Process	GO:0006468	protein phosphorylation	2/112	108/3002	0.99802	2
Biological Process	GO:0034654	nucleobase-containing compound biosynthetic process	2/112	110/3002	0.99802	2
Biological Process	GO:0060255	regulation of macromolecule metabolic process	6/112	254/3002	0.99802	6
Biological Process	GO:0019222	regulation of metabolic process	6/112	255/3002	0.99802	6
Biological Process	GO:0019438	aromatic compound biosynthetic process	2/112	111/3002	0.99802	2
Biological Process	GO:0018130	heterocycle biosynthetic process	2/112	113/3002	0.99802	2
Biological Process	GO:0044248	cellular catabolic process	1/112	70/3002	0.99802	1
Biological Process	GO:1901362	organic cyclic compound biosynthetic process	2/112	114/3002	0.99802	2
Biological Process	GO:0006508	proteolysis	3/112	154/3002	0.99802	3
Biological Process	GO:0046907	intracellular transport	2/112	115/3002	0.99802	2
Biological Process	GO:1901564	organonitrogen compound metabolic process	26/112	884/3002	0.99802	26
Biological Process	GO:0050790	regulation of catalytic activity	1/112	76/3002	0.99802	1
Biological Process	GO:0065009	regulation of molecular function	1/112	76/3002	0.99802	1
Biological Process	GO:0009056	catabolic process	3/112	164/3002	0.99802	3
Biological Process	GO:0031323	regulation of cellular metabolic process	5/112	238/3002	0.99802	5
Biological	GO:002260	cellular component assembly	2/112	125/300	0.99802	2

Process	7			2		
Biological Process	GO:0008380	RNA splicing	1/112	80/3002	0.99802	1
Biological Process	GO:0034622	cellular protein-containing complex assembly	1/112	84/3002	0.99802	1
Biological Process	GO:0007018	microtubule-based movement	1/112	86/3002	0.99802	1
Biological Process	GO:0090304	nucleic acid metabolic process	8/112	355/3002	0.99802	8
Biological Process	GO:0016192	vesicle-mediated transport	1/112	88/3002	0.99802	1
Biological Process	GO:0015031	protein transport	2/112	142/3002	0.99802	2
Biological Process	GO:0015833	peptide transport	2/112	142/3002	0.99802	2
Biological Process	GO:0042886	amide transport	2/112	142/3002	0.99802	2
Biological Process	GO:0045184	establishment of protein localization	2/112	142/3002	0.99802	2
Biological Process	GO:0065003	protein-containing complex assembly	1/112	94/3002	0.99802	1
Biological Process	GO:0008104	protein localization	2/112	144/3002	0.99802	2
Biological Process	GO:0033036	macromolecule localization	2/112	144/3002	0.99802	2
Biological Process	GO:0006928	movement of cell or subcellular component	1/112	97/3002	0.99802	1
Biological Process	GO:0043933	protein-containing complex subunit organization	1/112	97/3002	0.99802	1
Biological Process	GO:0071705	nitrogen compound transport	2/112	154/3002	0.99802	2
Biological Process	GO:0050789	regulation of biological process	10/112	456/3002	0.99802	10
Biological Process	GO:0006139	nucleobase-containing compound metabolic process	11/112	497/3002	0.99802	11
Biological Process	GO:0071702	organic substance transport	2/112	162/3002	0.99802	2
Biological Process	GO:0006725	cellular aromatic compound metabolic process	11/112	501/3002	0.99802	11
Biological Process	GO:0046483	heterocycle metabolic process	11/112	501/3002	0.99802	11
Biological Process	GO:0050794	regulation of cellular process	9/112	435/3002	0.99802	9
Biological	GO:190136	organic cyclic compound metabolic	11/11	507/300	0.99802	11

Process	0	process	2	2		
Biological Process	GO:0006464	cellular protein modification process	3/112	284/3002	0.999284	3
Biological Process	GO:0036211	protein modification process	3/112	284/3002	0.999284	3
Biological Process	GO:0043412	macromolecule modification	3/112	294/3002	0.999284	3
Cellular Component	GO:0043228	non-membrane-bounded organelle	32/85	490/2246	0.014685	32
Cellular Component	GO:0043232	intracellular non-membrane-bounded organelle	32/85	490/2246	0.014685	32
Cellular Component	GO:0005856	cytoskeleton	20/85	252/2246	0.014685	20
Cellular Component	GO:0070469	respirasome	5/85	23/2246	0.014685	5
Cellular Component	GO:0005874	microtubule	16/85	189/2246	0.014685	16
Cellular Component	GO:0099080	supramolecular complex	16/85	191/2246	0.014685	16
Cellular Component	GO:0099081	supramolecular polymer	16/85	191/2246	0.014685	16
Cellular Component	GO:0099512	supramolecular fiber	16/85	191/2246	0.014685	16
Cellular Component	GO:0099513	polymeric cytoskeletal fiber	16/85	191/2246	0.014685	16
Cellular Component	GO:0031966	mitochondrial membrane	7/85	56/2246	0.036558	7
Cellular Component	GO:0005743	mitochondrial inner membrane	6/85	54/2246	0.101072	6
Cellular Component	GO:0019866	organelle inner membrane	6/85	54/2246	0.101072	6
Cellular Component	GO:0005623	cell	7/85	76/2246	0.135296	7
Cellular Component	GO:0005737	cytoplasm	28/85	520/2246	0.135296	28
Cellular Component	GO:0005788	endoplasmic reticulum lumen	3/85	18/2246	0.15217	3
Cellular Component	GO:0005739	mitochondrion	6/85	63/2246	0.15217	6
Cellular Component	GO:0031090	organelle membrane	9/85	133/2246	0.225573	9
Cellular Component	GO:0005849	mRNA cleavage factor complex	2/85	11/2246	0.225573	2
Cellular	GO:003197	membrane-enclosed lumen	3/85	25/2246	0.22557	3

Component	4				3	
Cellular Component	GO:0043233	organelle lumen	3/85	25/2246	0.225573	3
Cellular Component	GO:0070013	intracellular organelle lumen	3/85	25/2246	0.225573	3
Cellular Component	GO:0005840	ribosome	12/85	199/2246	0.225573	12
Cellular Component	GO:0016021	integral component of membrane	23/85	450/2246	0.225573	23
Cellular Component	GO:0031224	intrinsic component of membrane	23/85	450/2246	0.225573	23
Cellular Component	GO:0015935	small ribosomal subunit	3/85	26/2246	0.225573	3
Cellular Component	GO:0012507	ER to Golgi transport vesicle membrane	1/85	2/2246	0.225573	1
Cellular Component	GO:0030658	transport vesicle membrane	1/85	2/2246	0.225573	1
Cellular Component	GO:0005783	endoplasmic reticulum	5/85	63/2246	0.255137	5
Cellular Component	GO:0000243	commitment complex	1/85	3/2246	0.271692	1
Cellular Component	GO:0005684	U2-type spliceosomal complex	1/85	3/2246	0.271692	1
Cellular Component	GO:0030127	COPII vesicle coat	1/85	3/2246	0.271692	1
Cellular Component	GO:0071004	U2-type prespliceosome	1/85	3/2246	0.271692	1
Cellular Component	GO:0071010	prespliceosome	1/85	3/2246	0.271692	1
Cellular Component	GO:0016020	membrane	27/85	594/2246	0.377911	27
Cellular Component	GO:0000015	phosphopyruvate hydratase complex	2/85	22/2246	0.458422	2
Cellular Component	GO:0005815	microtubule organizing center	1/85	6/2246	0.458422	1
Cellular Component	GO:0022627	cytosolic small ribosomal subunit	1/85	6/2246	0.458422	1
Cellular Component	GO:0044391	ribosomal subunit	3/85	47/2246	0.564807	3
Cellular Component	GO:0005666	RNA polymerase III complex	1/85	9/2246	0.60228	1
Cellular Component	GO:0005736	RNA polymerase I complex	1/85	9/2246	0.60228	1
Cellular	GO:000042	DNA-directed RNA polymerase	1/85	11/2246	0.66068	1

Component	8	complex			2	
Cellular Component	GO:0005665	RNA polymerase II, core complex	1/85	11/2246	0.660682	1
Cellular Component	GO:0055029	nuclear DNA-directed RNA polymerase complex	1/85	11/2246	0.660682	1
Cellular Component	GO:0005685	U1 snRNP	1/85	12/2246	0.67661	1
Cellular Component	GO:0030880	RNA polymerase complex	1/85	12/2246	0.67661	1
Cellular Component	GO:0005789	endoplasmic reticulum membrane	2/85	35/2246	0.686051	2
Cellular Component	GO:0061695	transferase complex, transferring phosphorus-containing groups	1/85	15/2246	0.768368	1
Cellular Component	GO:0033179	proton-transporting V-type ATPase, V0 domain	1/85	18/2246	0.854419	1
Cellular Component	GO:0005885	Arp2/3 protein complex	1/85	19/2246	0.854419	1
Cellular Component	GO:0045261	proton-transporting ATP synthase complex, catalytic core F(1)	1/85	19/2246	0.854419	1
Cellular Component	GO:0097525	spliceosomal snRNP complex	1/85	20/2246	0.867061	1
Cellular Component	GO:0033177	proton-transporting two-sector ATPase complex, proton-transporting domain	1/85	22/2246	0.896049	1
Cellular Component	GO:0030532	small nuclear ribonucleoprotein complex	1/85	23/2246	0.896049	1
Cellular Component	GO:0120114	Sm-like protein family complex	1/85	23/2246	0.896049	1
Cellular Component	GO:0030660	Golgi-associated vesicle membrane	1/85	25/2246	0.909082	1
Cellular Component	GO:0030662	coated vesicle membrane	1/85	25/2246	0.909082	1
Cellular Component	GO:0033178	proton-transporting two-sector ATPase complex, catalytic domain	1/85	27/2246	0.934094	1
Cellular Component	GO:0012506	vesicle membrane	1/85	30/2246	0.939543	1
Cellular Component	GO:0030659	cytoplasmic vesicle membrane	1/85	30/2246	0.939543	1
Cellular Component	GO:0098805	whole membrane	1/85	32/2246	0.939543	1
Cellular Component	GO:0030120	vesicle coat	1/85	33/2246	0.939543	1
Cellular Component	GO:0000139	Golgi membrane	1/85	34/2246	0.939543	1

Cellular Component	GO:0031410	cytoplasmic vesicle	1/85	34/2246	0.939543	1
Cellular Component	GO:0097708	intracellular vesicle	1/85	34/2246	0.939543	1
Cellular Component	GO:0031982	vesicle	1/85	36/2246	0.946587	1
Cellular Component	GO:0005852	eukaryotic translation initiation factor 3 complex	1/85	39/2246	0.946587	1
Cellular Component	GO:1990904	ribonucleoprotein complex	4/85	140/2246	0.946587	4
Cellular Component	GO:0005681	spliceosomal complex	1/85	43/2246	0.946587	1
Cellular Component	GO:0098588	bounding membrane of organelle	1/85	44/2246	0.946587	1
Cellular Component	GO:0043231	intracellular membrane-bounded organelle	19/85	598/2246	0.946587	19
Cellular Component	GO:0016459	myosin complex	2/85	90/2246	0.946587	2
Cellular Component	GO:0043227	membrane-bounded organelle	19/85	607/2246	0.946587	19
Cellular Component	GO:0098796	membrane protein complex	3/85	129/2246	0.946587	3
Cellular Component	GO:0000502	proteasome complex	1/85	57/2246	0.946587	1
Cellular Component	GO:0030117	membrane coat	1/85	57/2246	0.946587	1
Cellular Component	GO:1905369	endopeptidase complex	1/85	57/2246	0.946587	1
Cellular Component	GO:0005875	microtubule associated complex	1/85	59/2246	0.946587	1
Cellular Component	GO:1905368	peptidase complex	1/85	59/2246	0.946587	1
Cellular Component	GO:1990234	transferase complex	1/85	65/2246	0.956509	1
Cellular Component	GO:1902494	catalytic complex	4/85	227/2246	0.999022	4
Cellular Component	GO:0005634	nucleus	8/85	433/2246	0.999022	8
Cellular Component	GO:0032991	protein-containing complex	18/85	804/2246	0.999022	18
Molecular Function	GO:0005198	structural molecule activity	27/133	376/3612	0.015966	27
Molecular Function	GO:0004322	ferroxidase activity	5/133	21/3612	0.015966	5

Molecular Function	GO:0008199	ferric iron binding	5/133	21/3612	0.015966	5
Molecular Function	GO:0016722	oxidoreductase activity, oxidizing metal ions	5/133	21/3612	0.015966	5
Molecular Function	GO:0016724	oxidoreductase activity, oxidizing metal ions, oxygen as acceptor	5/133	21/3612	0.015966	5
Molecular Function	GO:0005200	structural constituent of cytoskeleton	15/133	167/3612	0.015966	15
Molecular Function	GO:0001883	purine nucleoside binding	28/133	420/3612	0.015966	28
Molecular Function	GO:0005525	GTP binding	28/133	420/3612	0.015966	28
Molecular Function	GO:0019001	guanyl nucleotide binding	28/133	420/3612	0.015966	28
Molecular Function	GO:0032550	purine ribonucleoside binding	28/133	420/3612	0.015966	28
Molecular Function	GO:0032561	guanyl ribonucleotide binding	28/133	420/3612	0.015966	28
Molecular Function	GO:0001882	nucleoside binding	28/133	426/3612	0.016881	28
Molecular Function	GO:0032549	ribonucleoside binding	28/133	426/3612	0.016881	28
Molecular Function	GO:0005506	iron ion binding	5/133	31/3612	0.058464	5
Molecular Function	GO:0003924	GTPase activity	19/133	289/3612	0.09495	19
Molecular Function	GO:0003954	NADH dehydrogenase activity	4/133	25/3612	0.10641	4
Molecular Function	GO:0008137	NADH dehydrogenase (ubiquinone) activity	4/133	25/3612	0.10641	4
Molecular Function	GO:0016655	oxidoreductase activity, acting on NAD(P)H, quinone or similar compound as acceptor	4/133	25/3612	0.10641	4
Molecular Function	GO:0050136	NADH dehydrogenase (quinone) activity	4/133	25/3612	0.10641	4
Molecular Function	GO:0015293	symporter activity	3/133	14/3612	0.109463	3
Molecular Function	GO:0004611	phosphoenolpyruvate carboxykinase activity	7/133	72/3612	0.124252	7
Molecular Function	GO:0016831	carboxy-lyase activity	7/133	73/3612	0.127269	7
Molecular Function	GO:0003735	structural constituent of ribosome	12/133	174/3612	0.173182	12
Molecular	GO:001529	secondary active transmembrane	4/133	31/3612	0.17318	4

Function	1	transporter activity			2	
Molecular Function	GO:0016829	lyase activity	11/133	155/3612	0.173182	11
Molecular Function	GO:0004587	ornithine-oxo-acid transaminase activity	1/133	1/3612	0.212091	1
Molecular Function	GO:0005326	neurotransmitter transmembrane transporter activity	2/133	9/3612	0.212091	2
Molecular Function	GO:0005328	neurotransmitter:sodium symporter activity	2/133	9/3612	0.212091	2
Molecular Function	GO:0008483	transaminase activity	2/133	9/3612	0.212091	2
Molecular Function	GO:0015294	solute:cation symporter activity	2/133	9/3612	0.212091	2
Molecular Function	GO:0015370	solute:sodium symporter activity	2/133	9/3612	0.212091	2
Molecular Function	GO:0016769	transferase activity, transferring nitrogenous groups	2/133	9/3612	0.212091	2
Molecular Function	GO:0015081	sodium ion transmembrane transporter activity	2/133	10/3612	0.242704	2
Molecular Function	GO:0022857	transmembrane transporter activity	11/133	172/3612	0.242704	11
Molecular Function	GO:0016830	carbon-carbon lyase activity	7/133	92/3612	0.242704	7
Molecular Function	GO:0030170	pyridoxal phosphate binding	3/133	25/3612	0.264849	3
Molecular Function	GO:0070279	vitamin B6 binding	3/133	25/3612	0.264849	3
Molecular Function	GO:0016835	carbon-oxygen lyase activity	4/133	41/3612	0.264849	4
Molecular Function	GO:0016836	hydro-lyase activity	4/133	41/3612	0.264849	4
Molecular Function	GO:0005215	transporter activity	11/133	180/3612	0.271461	11
Molecular Function	GO:0004831	tyrosine-tRNA ligase activity	1/133	2/3612	0.292716	1
Molecular Function	GO:0019842	vitamin binding	3/133	27/3612	0.296545	3
Molecular Function	GO:0016491	oxidoreductase activity	15/133	278/3612	0.323554	15
Molecular Function	GO:0016627	oxidoreductase activity, acting on the CH-CH group of donors	3/133	30/3612	0.363265	3
Molecular Function	GO:0004613	phosphoenolpyruvate carboxykinase (GTP) activity	2/133	15/3612	0.381378	2
Molecular	GO:000470	G protein-coupled receptor kinase	1/133	3/3612	0.38423	1

Function	3	activity			9	
Molecular Function	GO:0022804	active transmembrane transporter activity	4/133	51/3612	0.411659	4
Molecular Function	GO:0003729	mRNA binding	3/133	33/3612	0.413937	3
Molecular Function	GO:0003994	aconitate hydratase activity	2/133	17/3612	0.432835	2
Molecular Function	GO:0004466	long-chain-acyl-CoA dehydrogenase activity	1/133	4/3612	0.449266	1
Molecular Function	GO:0070840	dynein complex binding	1/133	4/3612	0.449266	1
Molecular Function	GO:0015077	monovalent inorganic cation transmembrane transporter activity	5/133	75/3612	0.449266	5
Molecular Function	GO:0004108	citrate (Si)-synthase activity	2/133	20/3612	0.511675	2
Molecular Function	GO:0036440	citrate synthase activity	2/133	20/3612	0.511675	2
Molecular Function	GO:0004827	proline-tRNA ligase activity	1/133	5/3612	0.516496	1
Molecular Function	GO:0017111	nucleoside-triphosphatase activity	26/133	591/3612	0.531591	26
Molecular Function	GO:0022853	active ion transmembrane transporter activity	3/133	41/3612	0.531591	3
Molecular Function	GO:0004634	phosphopyruvate hydratase activity	2/133	22/3612	0.531591	2
Molecular Function	GO:0016462	pyrophosphatase activity	26/133	597/3612	0.531591	26
Molecular Function	GO:0016818	hydrolase activity, acting on acid anhydrides, in phosphorus-containing anhydrides	26/133	597/3612	0.531591	26
Molecular Function	GO:0016651	oxidoreductase activity, acting on NAD(P)H	4/133	63/3612	0.531591	4
Molecular Function	GO:0030619	U1 snRNA binding	1/133	6/3612	0.531591	1
Molecular Function	GO:0016817	hydrolase activity, acting on acid anhydrides	26/133	598/3612	0.531591	26
Molecular Function	GO:0046912	transferase activity, transferring acyl groups, acyl groups converted into alkyl on transfer	2/133	23/3612	0.536459	2
Molecular Function	GO:0015318	inorganic molecular entity transmembrane transporter activity	7/133	132/3612	0.540034	7
Molecular Function	GO:0015075	ion transmembrane transporter activity	7/133	133/3612	0.545346	7
Molecular	GO:005066	coenzyme binding	5/133	89/3612	0.55606	5

Function	2				4	
Molecular Function	GO:0004448	isocitrate dehydrogenase activity	1/133	7/3612	0.556064	1
Molecular Function	GO:0004450	isocitrate dehydrogenase (NADP+) activity	1/133	7/3612	0.556064	1
Molecular Function	GO:0022890	inorganic cation transmembrane transporter activity	5/133	90/3612	0.559301	5
Molecular Function	GO:0008324	cation transmembrane transporter activity	5/133	91/3612	0.567606	5
Molecular Function	GO:0050660	flavin adenine dinucleotide binding	2/133	26/3612	0.572032	2
Molecular Function	GO:0046872	metal ion binding	21/133	490/3612	0.585356	21
Molecular Function	GO:0046873	metal ion transmembrane transporter activity	2/133	27/3612	0.587633	2
Molecular Function	GO:0046914	transition metal ion binding	7/133	144/3612	0.595168	7
Molecular Function	GO:0051082	unfolded protein binding	6/133	120/3612	0.595168	6
Molecular Function	GO:0004129	cytochrome-c oxidase activity	1/133	9/3612	0.595168	1
Molecular Function	GO:0015002	heme-copper terminal oxidase activity	1/133	9/3612	0.595168	1
Molecular Function	GO:0016675	oxidoreductase activity, acting on a heme group of donors	1/133	9/3612	0.595168	1
Molecular Function	GO:0016676	oxidoreductase activity, acting on a heme group of donors, oxygen as acceptor	1/133	9/3612	0.595168	1
Molecular Function	GO:0048037	cofactor binding	8/133	171/3612	0.600773	8
Molecular Function	GO:0003995	acyl-CoA dehydrogenase activity	1/133	10/3612	0.633945	1
Molecular Function	GO:0000287	magnesium ion binding	2/133	31/3612	0.634458	2
Molecular Function	GO:0043169	cation binding	21/133	510/3612	0.638423	21
Molecular Function	GO:0003746	translation elongation factor activity	3/133	55/3612	0.640947	3
Molecular Function	GO:0004190	aspartic-type endopeptidase activity	1/133	11/3612	0.640947	1
Molecular Function	GO:0070001	aspartic-type peptidase activity	1/133	11/3612	0.640947	1
Molecular Function	GO:0000104	succinate dehydrogenase activity	1/133	12/3612	0.640947	1

Molecular Function	GO:0004111	creatine kinase activity	1/133	12/3612	0.640947	1
Molecular Function	GO:0008177	succinate dehydrogenase (ubiquinone) activity	1/133	12/3612	0.640947	1
Molecular Function	GO:0016635	oxidoreductase activity, acting on the CH-CH group of donors, quinone or related compound as acceptor	1/133	12/3612	0.640947	1
Molecular Function	GO:0016775	phosphotransferase activity, nitrogenous group as acceptor	1/133	12/3612	0.640947	1
Molecular Function	GO:0017069	snRNA binding	1/133	12/3612	0.640947	1
Molecular Function	GO:0020037	heme binding	1/133	12/3612	0.640947	1
Molecular Function	GO:0015078	proton transmembrane transporter activity	3/133	59/3612	0.648359	3
Molecular Function	GO:0046906	tetrapyrrole binding	1/133	13/3612	0.668291	1
Molecular Function	GO:0044877	protein-containing complex binding	4/133	88/3612	0.693057	4
Molecular Function	GO:0005452	inorganic anion exchanger activity	1/133	14/3612	0.693057	1
Molecular Function	GO:0051015	actin filament binding	3/133	64/3612	0.706629	3
Molecular Function	GO:0004829	threonine-tRNA ligase activity	1/133	15/3612	0.715451	1
Molecular Function	GO:0003723	RNA binding	17/133	435/3612	0.715823	17
Molecular Function	GO:0004812	aminoacyl-tRNA ligase activity	4/133	93/3612	0.724956	4
Molecular Function	GO:0016875	ligase activity, forming carbon-oxygen bonds	4/133	93/3612	0.724956	4
Molecular Function	GO:0140101	catalytic activity, acting on a tRNA	4/133	94/3612	0.730394	4
Molecular Function	GO:0015103	inorganic anion transmembrane transporter activity	2/133	42/3612	0.730394	2
Molecular Function	GO:0019843	rRNA binding	1/133	17/3612	0.735846	1
Molecular Function	GO:0008509	anion transmembrane transporter activity	2/133	43/3612	0.735846	2
Molecular Function	GO:0051539	4 iron, 4 sulfur cluster binding	2/133	44/3612	0.742206	2
Molecular Function	GO:0015252	proton channel activity	1/133	18/3612	0.742206	1
Molecular	GO:004693	proton-transporting ATP synthase	1/133	18/3612	0.74220	1

Function	3	activity, rotational mechanism			6	
Molecular Function	GO:0016301	kinase activity	9/133	235/3612	0.751319	9
Molecular Function	GO:0016772	transferase activity, transferring phosphorus-containing groups	11/133	292/3612	0.76428	11
Molecular Function	GO:0016787	hydrolase activity	31/133	842/3612	0.786067	31
Molecular Function	GO:0008135	translation factor activity, RNA binding	5/133	133/3612	0.786113	5
Molecular Function	GO:0090079	translation regulator activity, nucleic acid binding	5/133	133/3612	0.786113	5
Molecular Function	GO:0004386	helicase activity	3/133	78/3612	0.786113	3
Molecular Function	GO:0016887	ATPase activity	5/133	134/3612	0.786113	5
Molecular Function	GO:0045182	translation regulator activity	5/133	136/3612	0.791663	5
Molecular Function	GO:0140098	catalytic activity, acting on RNA	5/133	136/3612	0.791663	5
Molecular Function	GO:0016776	phosphotransferase activity, phosphate group as acceptor	1/133	24/3612	0.822785	1
Molecular Function	GO:0005261	cation channel activity	1/133	27/3612	0.858497	1
Molecular Function	GO:0015114	phosphate ion transmembrane transporter activity	1/133	27/3612	0.858497	1
Molecular Function	GO:0015321	sodium-dependent phosphate transmembrane transporter activity	1/133	27/3612	0.858497	1
Molecular Function	GO:0005216	ion channel activity	1/133	28/3612	0.858497	1
Molecular Function	GO:0015267	channel activity	1/133	28/3612	0.858497	1
Molecular Function	GO:0022803	passive transmembrane transporter activity	1/133	28/3612	0.858497	1
Molecular Function	GO:0016616	oxidoreductase activity, acting on the CH-OH group of donors, NAD or NADP as acceptor	1/133	29/3612	0.859419	1
Molecular Function	GO:0005509	calcium ion binding	4/133	123/3612	0.859419	4
Molecular Function	GO:0009055	electron transfer activity	1/133	30/3612	0.859419	1
Molecular Function	GO:0003700	DNA-binding transcription factor activity	3/133	95/3612	0.859419	3
Molecular Function	GO:0042623	ATPase activity, coupled	3/133	95/3612	0.859419	3

Molecular Function	GO:0051536	iron-sulfur cluster binding	2/133	66/3612	0.859419	2
Molecular Function	GO:0051540	metal cluster binding	2/133	66/3612	0.859419	2
Molecular Function	GO:0003899	DNA-directed 5'-3' RNA polymerase activity	1/133	33/3612	0.859419	1
Molecular Function	GO:0016614	oxidoreductase activity, acting on CH-OH group of donors	1/133	33/3612	0.859419	1
Molecular Function	GO:0034062	5'-3' RNA polymerase activity	1/133	33/3612	0.859419	1
Molecular Function	GO:0097747	RNA polymerase activity	1/133	33/3612	0.859419	1
Molecular Function	GO:0008234	cysteine-type peptidase activity	2/133	67/3612	0.859419	2
Molecular Function	GO:0008144	drug binding	30/133	888/3612	0.885022	30
Molecular Function	GO:0003779	actin binding	4/133	140/3612	0.904073	4
Molecular Function	GO:0016746	transferase activity, transferring acyl groups	2/133	75/3612	0.904073	2
Molecular Function	GO:0016740	transferase activity	17/133	531/3612	0.904073	17
Molecular Function	GO:0003743	translation initiation factor activity	2/133	77/3612	0.90987	2
Molecular Function	GO:0016757	transferase activity, transferring glycosyl groups	1/133	43/3612	0.925295	1
Molecular Function	GO:0004175	endopeptidase activity	1/133	45/3612	0.93544	1
Molecular Function	GO:0140110	transcription regulator activity	3/133	121/3612	0.935694	3
Molecular Function	GO:0070011	peptidase activity, acting on L-amino acid peptides	3/133	122/3612	0.935694	3
Molecular Function	GO:0016874	ligase activity	4/133	158/3612	0.935694	4
Molecular Function	GO:0016779	nucleotidyltransferase activity	1/133	50/3612	0.935694	1
Molecular Function	GO:0005524	ATP binding	27/133	862/3612	0.935694	27
Molecular Function	GO:0030554	adenyl nucleotide binding	27/133	862/3612	0.935694	27
Molecular Function	GO:0032559	adenyl ribonucleotide binding	27/133	862/3612	0.935694	27
Molecular Function	GO:0008233	peptidase activity	3/133	129/3612	0.935694	3

Molecular Function	GO:0030234	enzyme regulator activity	1/133	58/3612	0.953333	1
Molecular Function	GO:0008270	zinc ion binding	2/133	100/3612	0.953333	2
Molecular Function	GO:0008092	cytoskeletal protein binding	4/133	178/3612	0.959861	4
Molecular Function	GO:0003676	nucleic acid binding	24/133	811/3612	0.960344	24
Molecular Function	GO:0004672	protein kinase activity	2/133	108/3612	0.960344	2
Molecular Function	GO:0043565	sequence-specific DNA binding	1/133	76/3612	0.975459	1
Molecular Function	GO:0098772	molecular function regulator	1/133	76/3612	0.975459	1
Molecular Function	GO:0004674	protein serine/threonine kinase activity	1/133	77/3612	0.975459	1
Molecular Function	GO:0016773	phosphotransferase activity, alcohol group as acceptor	2/133	130/3612	0.98028	2
Molecular Function	GO:0005515	protein binding	10/133	427/3612	0.98028	10
Molecular Function	GO:0003677	DNA binding	4/133	238/3612	0.991841	4
Molecular Function	GO:0003774	motor activity	2/133	168/3612	0.993818	2
Molecular Function	GO:0140096	catalytic activity, acting on a protein	5/133	328/3612	0.995135	5

Table S2. KEGG enrichment analysis of DEGs at level-3.

Pathway ID	Pathways	GeneRatio	BgRatio	q-value	Count
ko04145	Phagosome	24/99	291/2589	0.024394	24
ko04976	Bile secretion	4/99	15/2589	0.069624	4
ko04540	Gap junction	15/99	175/2589	0.069624	15
ko04964	Proximal tubule bicarbonate reclamation	6/99	37/2589	0.069624	6
ko04978	Mineral absorption	5/99	26/2589	0.069624	5
ko04974	Protein digestion and absorption	4/99	17/2589	0.073082	4
ko04972	Pancreatic secretion	6/99	40/2589	0.073082	6

ko04973	Carbohydrate digestion and absorption	4/99	18/2589	0.073754	4
ko04911	Insulin secretion	4/99	20/2589	0.099099	4
ko04960	Aldosterone-regulated sodium reabsorption	4/99	22/2589	0.128268	4
ko04919	Thyroid hormone signaling pathway	8/99	81/2589	0.149377	8
ko04961	Endocrine and other factor-regulated calcium reabsorption	4/99	28/2589	0.245218	4
ko04918	Thyroid hormone synthesis	5/99	43/2589	0.245218	5
ko03015	mRNA surveillance pathway	11/99	147/2589	0.245218	11
ko04530	Tight junction	13/99	190/2589	0.256842	13
ko03018	RNA degradation	8/99	95/2589	0.256842	8
ko04970	Salivary secretion	4/99	35/2589	0.35773	4
ko05100	Bacterial invasion of epithelial cells	7/99	86/2589	0.35773	7
ko04210	Apoptosis	11/99	164/2589	0.35773	11
ko04971	Gastric acid secretion	4/99	36/2589	0.35773	4
ko03010	Ribosome	12/99	186/2589	0.35773	12
ko05131	Shigellosis	7/99	90/2589	0.382169	7
ko05205	Proteoglycans in cancer	9/99	130/2589	0.387655	9
ko04925	Aldosterone synthesis and secretion	4/99	39/2589	0.387655	4
ko05416	Viral myocarditis	7/99	95/2589	0.428828	7
ko05132	Salmonella infection	7/99	96/2589	0.431976	7
ko00514	Other types of O-glycan biosynthesis	1/99	2/2589	0.436778	1
ko00020	Citrate cycle (TCA cycle)	7/99	99/2589	0.437308	7
ko04670	Leukocyte transendothelial migration	6/99	80/2589	0.437308	6

ko05412	Arrhythmogenic right ventricular cardiomyopathy (ARVC)	4/99	44/2589	0.437308	4
ko05014	Amyotrophic lateral sclerosis (ALS)	2/99	13/2589	0.437308	2
ko05418	Fluid shear stress and atherosclerosis	9/99	142/2589	0.439188	9
ko04714	Thermogenesis	8/99	122/2589	0.439188	8
ko04612	Antigen processing and presentation	7/99	104/2589	0.465078	7
ko04260	Cardiac muscle contraction	5/99	66/2589	0.470528	5
ko05032	Morphine addiction	2/99	15/2589	0.470528	2
ko04742	Taste transduction	1/99	3/2589	0.470528	1
ko04151	PI3K-Akt signaling pathway	9/99	149/2589	0.470528	9
ko04650	Natural killer cell mediated cytotoxicity	2/99	16/2589	0.490581	2
ko04024	cAMP signaling pathway	6/99	90/2589	0.490581	6
ko05410	Hypertrophic cardiomyopathy (HCM)	4/99	52/2589	0.490581	4
ko05414	Dilated cardiomyopathy (DCM)	4/99	52/2589	0.490581	4
ko00720	Carbon fixation pathways in prokaryotes	3/99	34/2589	0.490581	3
ko05142	Chagas disease (American trypanosomiasis)	4/99	53/2589	0.490581	4
ko00565	Ether lipid metabolism	1/99	4/2589	0.490581	1
ko00860	Porphyrin and chlorophyll metabolism	1/99	4/2589	0.490581	1
ko04940	Type I diabetes mellitus	1/99	4/2589	0.490581	1
ko04810	Regulation of actin cytoskeleton	7/99	115/2589	0.490581	7
ko04015	Rap1 signaling pathway	6/99	96/2589	0.491161	6
ko04520	Adherens junction	6/99	96/2589	0.491161	6
ko04380	Osteoclast differentiation	2/99	19/2589	0.491161	2

ko04620	Toll-like receptor signaling pathway	2/99	19/2589	0.491161	2
ko04662	B cell receptor signaling pathway	2/99	19/2589	0.491161	2
ko04666	Fc gamma R-mediated phagocytosis	3/99	38/2589	0.513777	3
ko01521	EGFR tyrosine kinase inhibitor resistance	2/99	20/2589	0.513777	2
ko03013	RNA transport	9/99	167/2589	0.528593	9
ko04664	Fc epsilon RI signaling pathway	2/99	21/2589	0.531385	2
ko04745	Phototransduction - fly	4/99	60/2589	0.531385	4
ko04391	Hippo signaling pathway - fly	5/99	81/2589	0.531385	5
ko05231	Choline metabolism in cancer	2/99	22/2589	0.546732	2
ko04141	Protein processing in endoplasmic reticulum	10/99	196/2589	0.558463	10
ko04510	Focal adhesion	6/99	107/2589	0.568662	6
ko05163	Human cytomegalovirus infection	5/99	85/2589	0.568662	5
ko04217	Necroptosis	6/99	110/2589	0.607499	6
ko04370	VEGF signaling pathway	2/99	25/2589	0.611319	2
ko04022	cGMP-PKG signaling pathway	5/99	91/2589	0.62774	5
ko00471	D-Glutamine and D-glutamate metabolism	1/99	8/2589	0.62774	1
ko05164	Influenza A	6/99	114/2589	0.62774	6
ko00630	Glyoxylate and dicarboxylate metabolism	4/99	69/2589	0.62774	4
ko05134	Legionellosis	4/99	70/2589	0.63873	4
ko04740	Olfactory transduction	2/99	28/2589	0.652741	2
ko04071	Sphingolipid signaling pathway	4/99	72/2589	0.652741	4
ko00600	Sphingolipid metabolism	1/99	9/2589	0.652741	1

ko05212	Pancreatic cancer	2/99	29/258 9	0.6631 38	2
ko05170	Human immunodeficiency virus 1 infection	5/99	100/25 89	0.7145 76	5
ko04062	Chemokine signaling pathway	3/99	54/258 9	0.7145 76	3
ko05166	Human T-cell leukemia virus 1 infection	4/99	78/258 9	0.7145 76	4
ko00480	Glutathione metabolism	1/99	11/258 9	0.7145 76	1
ko04390	Hippo signaling pathway	5/99	102/25 89	0.7145 76	5
ko05225	Hepatocellular carcinoma	4/99	79/258 9	0.7192 65	4
ko05110	Vibrio cholerae infection	5/99	105/25 89	0.7279 97	5
ko04216	Ferroptosis	1/99	12/258 9	0.7279 97	1
ko04611	Platelet activation	4/99	81/258 9	0.7279 97	4
ko04933	AGE-RAGE signaling pathway in diabetic complications	2/99	35/258 9	0.7386 86	2
ko05210	Colorectal cancer	2/99	35/258 9	0.7386 86	2
ko04261	Adrenergic signaling in cardiomyocytes	6/99	133/25 89	0.7413 21	6
ko04066	HIF-1 signaling pathway	4/99	84/258 9	0.7413 21	4
ko04659	Th17 cell differentiation	3/99	60/258 9	0.7413 21	3
ko04657	IL-17 signaling pathway	3/99	61/258 9	0.7520 03	3
ko04932	Non-alcoholic fatty liver disease (NAFLD)	3/99	63/258 9	0.7808 51	3
ko05120	Epithelial cell signaling in Helicobacter pylori infection	2/99	40/258 9	0.8006 62	2
ko00071	Fatty acid degradation	1/99	16/258 9	0.8006 62	1
ko04626	Plant-pathogen interaction	3/99	66/258 9	0.8006 62	3
ko04921	Oxytocin signaling pathway	5/99	117/25 89	0.8006 62	5
ko05162	Measles	2/99	42/258 9	0.8189 9	2

ko00680	Methane metabolism	2/99	43/2589	0.823124	2
ko04931	Insulin resistance	2/99	43/2589	0.823124	2
ko04914	Progesterone-mediated oocyte maturation	3/99	71/2589	0.831118	3
ko04020	Calcium signaling pathway	2/99	45/2589	0.831118	2
ko04360	Axon guidance	2/99	45/2589	0.831118	2
ko04623	Cytosolic DNA-sensing pathway	1/99	19/2589	0.831118	1
ko00970	Aminoacyl-tRNA biosynthesis	3/99	72/2589	0.831118	3
ko05202	Transcriptional misregulation in cancer	1/99	20/2589	0.831118	1
ko05211	Renal cell carcinoma	2/99	47/2589	0.831118	2
ko04910	Insulin signaling pathway	3/99	75/2589	0.831118	3
ko05215	Prostate cancer	3/99	75/2589	0.831118	3
ko04920	Adipocytokine signaling pathway	1/99	21/2589	0.831118	1
ko04915	Estrogen signaling pathway	4/99	103/2589	0.831118	4
ko04010	MAPK signaling pathway	3/99	76/2589	0.831118	3
ko04150	mTOR signaling pathway	2/99	49/2589	0.831118	2
ko04621	NOD-like receptor signaling pathway	3/99	77/2589	0.831919	3
ko04341	Hedgehog signaling pathway - fly	1/99	22/2589	0.832884	1
ko04152	AMPK signaling pathway	3/99	79/2589	0.8415	3
ko04924	Renin secretion	1/99	23/2589	0.8415	1
ko00910	Nitrogen metabolism	1/99	24/2589	0.856273	1
ko00220	Arginine biosynthesis	1/99	26/2589	0.873715	1
ko00760	Nicotinate and nicotinamide metabolism	1/99	26/2589	0.873715	1

ko04013	MAPK signaling pathway - fly	2/99	57/258 9	0.8737 15	2
ko04146	Peroxisome	1/99	27/258 9	0.8737 15	1
ko05012	Parkinson disease	3/99	88/258 9	0.8737 15	3
ko00250	Alanine, aspartate and glutamate metabolism	1/99	28/258 9	0.8737 15	1
ko00230	Purine metabolism	2/99	60/258 9	0.8737 15	2
ko04371	Apelin signaling pathway	2/99	60/258 9	0.8737 15	2
ko03020	RNA polymerase	1/99	29/258 9	0.8737 15	1
ko05020	Prion diseases	1/99	29/258 9	0.8737 15	1
ko03040	Spliceosome	7/99	205/25 89	0.8737 15	7
ko04144	Endocytosis	4/99	124/25 89	0.8783 39	4
ko03320	PPAR signaling pathway	2/99	64/258 9	0.8783 39	2
ko04214	Apoptosis - fly	1/99	32/258 9	0.8783 39	1
ko04218	Cellular senescence	2/99	65/258 9	0.8783 39	2
ko00620	Pyruvate metabolism	1/99	33/258 9	0.8783 39	1
ko04730	Long-term depression	1/99	33/258 9	0.8783 39	1
ko04014	Ras signaling pathway	2/99	66/258 9	0.8783 39	2
ko04722	Neurotrophin signaling pathway	2/99	66/258 9	0.8783 39	2
ko00010	Glycolysis / Gluconeogenesis	3/99	99/258 9	0.8869 37	3
ko04213	Longevity regulating pathway - multiple species	1/99	35/258 9	0.8886 07	1
ko05167	Kaposi sarcoma-associated herpesvirus infection	2/99	70/258 9	0.8944 28	2
ko04212	Longevity regulating pathway - worm	1/99	37/258 9	0.8983 92	1
ko05160	Hepatitis C	1/99	39/258 9	0.9126 97	1

ko05200	Pathways in cancer	5/99	172/25<br>89	0.9185<br>53	5
ko05145	Toxoplasmosis	1/99	41/258<br>9	0.9185<br>53	1
ko04072	Phospholipase D signaling pathway	1/99	42/258<br>9	0.9185<br>53	1
ko00190	Oxidative phosphorylation	2/99	79/258<br>9	0.9185<br>53	2
ko04310	Wnt signaling pathway	2/99	80/258<br>9	0.9185<br>53	2
ko04138	Autophagy - yeast	1/99	44/258<br>9	0.9185<br>53	1
ko00240	Pyrimidine metabolism	1/99	45/258<br>9	0.9199<br>5	1
ko03060	Protein export	1/99	48/258<br>9	0.9348<br>04	1
ko05203	Viral carcinogenesis	2/99	90/258<br>9	0.9491<br>46	2
ko04113	Meiosis - yeast	1/99	55/258<br>9	0.9607<br>41	1
ko04350	TGF-beta signaling pathway	1/99	56/258<br>9	0.9607<br>41	1
ko04922	Glucagon signaling pathway	1/99	60/258<br>9	0.9656<br>92	1
ko05168	Herpes simplex infection	1/99	62/258<br>9	0.9656<br>92	1
ko04111	Cell cycle - yeast	1/99	65/258<br>9	0.9656<br>92	1
ko05010	Alzheimer disease	2/99	107/25<br>89	0.9656<br>92	2
ko04728	Dopaminergic synapse	1/99	69/258<br>9	0.9656<br>92	1
ko05152	Tuberculosis	1/99	70/258<br>9	0.9656<br>92	1
ko05016	Huntington disease	4/99	189/25<br>89	0.9656<br>92	4
ko04068	FoxO signaling pathway	1/99	72/258<br>9	0.9656<br>92	1
ko04114	Oocyte meiosis	1/99	85/258<br>9	0.9755<br>21	1
ko05169	Epstein-Barr virus infection	3/99	173/25<br>89	0.9755<br>21	3
ko03050	Proteasome	1/99	88/258<br>9	0.9755<br>21	1

ko05165	Human papillomavirus infection	1/99	112/2589	0.988497	1

Table S3. Information of primers.

Gene name	Primer sequence		TM (°C)	Product length (bp)
EF1α	Forward	TGCTGGTACTGGTGAGTTCG	59.68	238
	Reverse	CCATCCAGAGATTGGCACGA	59.82	
RBM25	Forward	CAGATTGCGAGAGCGACAGA	60.18	147
	Reverse	CTCTCGCGCTCAATCTTTGC	59.97	
DUOX	Forward	CAGTCAGCGGCTCTACGGTT	60.3	193
	Reverse	GGTTATCGTATGCGTCTGGTGA	60.2	
NNT	Forward	AGATTACTGGCACACACACGGA	60.4	292
	Reverse	TCAGTCTCTGGGAAGTCATCGT	60.4	
CHS1	Forward	GGATGGGATGAAACGCAAGC	59.9	121
	Reverse	CAACATCGCCATCCAAAGCC	60.18	
MEIS1	Forward	AGTGTGGTTTCTGTGCTTGTGG	60	160
	Reverse	CGTACATACTCTCCATGCCAGC	59.9	
metK	Forward	AAACCAGACACGGACCAATGA	60	123
	Reverse	TGGGCTGTTTTGCTGATGG	60	
TARS	Forward	AGGCGAGAATAAGGAGGCTGT	59.9	201
	Reverse	TCACGCTTCTTCTTTTCTGCC	59.9	
ABCC1	Forward	GGCGGATTCGAGGGGTATTT	59.89	132
	Reverse	GTACGAGTGAATTGCGGTGC	59.91	
